# Sex-Specific Signatures of Circulating Protein and Cellular Host Responses Predicting COVID-19 Severity

**DOI:** 10.3390/medsci14020282

**Published:** 2026-05-31

**Authors:** Milica Radisavljević, Zorica Stojić-Vukanić, Tijana Kosanović, Miodrag Lalošević, Iva Perović Blagojević, Jovana Milijić Jovanović, Aleksa Petković, Jelena Marjanović, Gordana Leposavić

**Affiliations:** 1 Department of Microbiology and Immunology, University of Belgrade—Faculty of Pharmacy, 11000 Belgrade, Serbia; milicas.ph@gmail.com (M.R.); zorica.stojic-vukanic@pharmacy.bg.ac.rs (Z.S.-V.); 2 Radiology Department, University Hospital “Dr Dragiša Mišović-Dedinje”, 11000 Belgrade, Serbia; tijanadrkosanovic@gmail.com (T.K.); dr.lalosevic@gmail.com (M.L.); 3 Department for Laboratory Diagnostic, University Hospital “Dr Dragiša Mišović-Dedinje”, 11000 Belgrade, Serbia; ivapb17@gmail.com (I.P.B.); jovana.d.jovanovic@outlook.com (J.M.J.); aleksa93@gmail.com (A.P.); jelenamarjanovic2702@gmail.com (J.M.); 4 Independent Researcher, 11000 Belgrade, Serbia

**Keywords:** sex-specificity of inflammatory response, acute-phase proteins, white blood cell counts, COVID-19 progression predictors

## Abstract

Background/Objectives: Although COVID-19 is generally more severe in males, data on sex-specific differences in the predictive value of commonly used inflammatory biomarkers remain limited. The study aimed to evaluate the sex-specific prognostic performance of selected biomarkers during the Alpha variant wave. Methods: In single-center study, univariate and multivariable regressions analyses, along with receiver operating characteristic curve (ROC) analyses, were performed to assess the association of acute-phase proteins, cytokines, and white blood cell indices (at admission and 7 days later) and disease severity and mortality in patients with severe-to-critical COVID-19. Results: At admission, the combined assessment of ferritin and D-dimer predicted disease severity in both sexes; however, optimal cut-off values and diagnostic performance (specificity and sensitivity) differed between males and females. In males, neutrophil and lymphocyte counts provided additional clinically relevant predictive value. Seven days after admission, the combination of ferritin, D-dimer, and fibrinogen in males, and ferritin, as an independent predictor within a model including lactate dehydrogenase, in females demonstrated strong predictive performance for severe-to-critical COVID-19. At this time-point, lymphocyte count in males was also identified as an independent predictor of disease severity. Notably, C-reactive protein and neutrophil count correlated with mortality in males with severe-to-critical disease. Conclusions: Severe COVID-19 is predicted by distinct acute-phase proteins and shared, sex-specific biomarkers, but with distinct cut-offs and predictive accuracy. In males, white blood cell indices also serve as independent predictors. Furthermore, prognostic utility changes of these biomarkers over the course of the disease, suggesting sex-specific and time-dependent role in COVID-19 pathogenesis.

## 1. Introduction

SARS-CoV-2 infection ranges from mild flu-like symptoms (cough, sore throat, myalgia, fatigue) to severe, critical, or fatal disease [1]. Severe cases feature tachypnea, dyspnea at rest or minimal activity, hemodynamic instability, and extensive chest X-ray infiltrates [1]. Elderly patients and individuals with comorbidities (such as hypertension, diabetes, and cardiovascular diseases) frequently develop critical illness requiring intensive care and mechanical ventilation [2].

Emerging in China in late 2019, SARS-CoV-2 spread rapidly, leading to a global pandemic by early 2020 [3]. The World Health Organization (WHO) declared a Public Health Emergency of International Concern (PHEIC) on 30 January 2020 [3] and characterized it as a pandemic on 11 March 2020. Although the global emergency officially ended in May 2023 [4], the virus has not been eradicated [5]. Consequently, new COVID-19 cases and fatalities persist globally, with surveillance data updating weekly to track transmission dynamics [6]. While emerging SARS-CoV-2 variants generally cause milder illness, high-risk populations (particularly the elderly and individuals with comorbidities) remain vulnerable to severe outcomes [7] However, Markov et al. [7] suggest this trend may be coincidental. Their analysis indicates that rapid viral evolution will likely produce new variants that are not only more threatening but also capable of evading immunity from full vaccination [7]. Furthermore, viral evolution could increase transmissivity, causing a sudden spike in COVID-19 cases and deaths [5]. As an obvious consequence of these facts, a custom PubMed query of all COVID-19-related MeSH terms and keywords revealed that tens of thousands, rather than hundreds of thousands, of new articles on different aspects of COVID-19 were added during the last 12 months.

Although the literature emphasizes the necessity of early prognostic risk factor identification for managing severe COVID-19, this remains a challenging clinical endeavor [8,9]. A severe systemic inflammatory response to SARS-CoV-2, often presenting as a cytokine storm, drives acute respiratory distress syndrome (ARDS), multi-organ dysfunction, and poor prognosis [10]. Inflammatory activity is, in part, indicated by elevated levels of C-reactive protein (CRP), ferritin, D-dimer, and lactate dehydrogenase (LDH), along with lymphopenia and neutrophilia [11,12,13,14,15,16,17]. These clinical indicators are vital for identifying, monitoring, and predicting disease severity to guide treatment strategies [11,12,13,14,15,16,17]. While COVID-19 severity biomarkers have been extensively researched, establishing validated, standardized correlations remains challenging. Furthermore, a critical lack of data persists regarding independent predictors and their integration into combined disease severity models.

Although all SARS-CoV-2 variants consistently exhibit sex-specific clinical disparities (with sex-specific disparities, notably worse outcomes among men), the underlying mechanisms driving these differences remain under-investigated [18,19,20]. Consequently, a critical need exists for sex-disaggregated data to elucidate how putative risk factors influence adverse COVID-19 outcomes. Given that inflammation is a central driver of severe disease [11], characterizing sex-specific host inflammatory profiles during hospitalization is essential to understanding the mechanisms underlying divergent clinical trajectories. Furthermore, defining putative sex-specific inflammatory predictors (including cut-off values, sensitivity, and specificity) of severe-to-critical disease is necessary to enhance risk stratification of COVID-19 patients. Moreover, such insights may also facilitate informed decision-making and the development of sex-specific tailored interventions for severe COVID-19 cases. 

Considering the above, this study investigated the qualitative and quantitative sex specificities of circulating inflammatory acute-phase proteins and immune cell-related signatures in the early phase of disease development. The goal was to define sex-specific risk models for severe disease progression and mortality. To identify potential predictors and risk models, longitudinal changes in inflammatory markers (acute-phase proteins, immune cells, and cytokines) were monitored at admission and 7 days later in COVID-19 patients admitted to “Dr Dragiša Mišović-Dedinje” hospital in Belgrade from September 2020 to April 2021 (corresponding mainly to the Alpha SARS-CoV-2 variant wave). We evaluated potential predictors using univariate and binary logistic regression, and assessed the predictive performance of the models using receiver operating characteristic (ROC) curve analysis.

## 2. Material and Methods

### 2.1. Study Design and Participants

This single-center retrospective study evaluated 87 adult COVID-19 patients admitted to the Clinical Hospital Center “Dr Dragiša Mišović-Dedinje” (Belgrade, Serbia) between September 2020 and April 2021. The study protocol adhered to the Declaration of Helsinki and was approved by the institutional Ethics Committee (No. 01-7661; 1 July 2020). All participants provided written informed consent prior to enrollment. Patients were informed of their right to withdraw at any time without penalty. Participation was voluntary and free of charge, with all study-related costs covered by the investigators. Strict data privacy and patient monitoring standards were maintained throughout the study.

**Figure 1:**
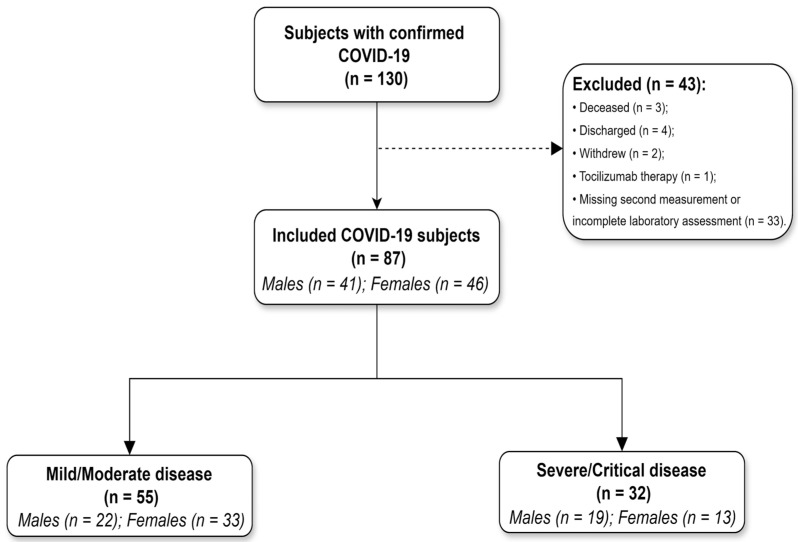
Flow diagram illustrates the selection of patients with confirmed COVID-19 for inclusion in the study. Patients who do not meet the study criteria are excluded from the pool.

The study initially enrolled 130 participants diagnosed with COVID-19 via clinical symptoms and RT-PCR testing [21]. We excluded 43 patients due to death (*n* = 3), hospital discharge (*n* = 4), withdrawal (*n* = 2), tocilizumab therapy (*n* = 1), or missing day-7 blood samples (*n* = 13). Additionally, 20 patients were excluded for missing baseline or day-7 laboratory results, specifically for LDH (*n* = 14), IL-6 (*n* = 3), ferritin (*n* = 2), and D-dimer (*n* = 1) (Figure 1). The remaining 87 participants completed follow-up with full laboratory data at admission and on day 7 after admission.

All patients underwent non-contrast chest computed tomography (CT) on the day of admission. All CT examinations were performed using a 320-row multidetector CT (MDCT) system (Aquilion ONE, TSX-301C; Canon Medical Systems, Tokyo, Japan) and interpreted by two experienced radiologists who were blinded to clinical and laboratory data. Discrepancies were resolved by consensus. The following parenchymal lung patterns were analyzed: ground-glass opacities, crazy-paving patterns, consolidation, and residual ground-glass opacities. The CT severity score was determined at admission to quantify pulmonary involvement. This score was calculated by visually estimating the percentage of involvement in each of the five lung lobes: 0 (none), 1 (<5%), 2 (5–25%), 3 (26–50%), 4 (51–75%), and 5 (>75%). Individual lobe scores were summed to generate a total score ranging from 0 to 25 at admission.

Upon admission, participants were stratified according to Institutes of Health (NIH) clinical spectrum guidelines for SARS-CoV-2 infection [22]. The cohort comprised 55 patients with mild-to-moderate illness and 32 patients with severe-to-critical illness. Mild-to-moderate disease (Mild/Moderate) was defined by a pulse oximetry oxygen saturation (SpO) ≥ 94% on room air at sea level, alongside the absence of abnormal chest imaging or clinical signs of lower respiratory tract disease. Conversely, severe-to-critical disease (Severe/Critical) was defined by a (SpO) < 94% on room air at sea level or the presence of manifest respiratory failure.

### 2.2. Data Collection

Participant data were retrieved from the hospital’s information management system and entered into a dedicated Excel database. The dataset included demographic characteristics (age and sex), comorbidities (cardiovascular diseases, diabetes mellitus, and cancer), CT severity scores, oxygen support requirements, administered therapies (favipiravir, corticosteroids, antibiotics, fraxiparine, and aspirin), and mortality outcomes. Laboratory data, including CRP, ferritin, fibrinogen, D-dimer, LDH, neutrophil and lymphocyte counts, IL-6, were assessed at admission and on day 7 after admission. Acute-phase proteins and immune cell-related blood indices were evaluated as predictors of severe COVID-19 or mortality.

#### 2.2.1. Blood Sample Collection and Processing

Venous blood samples were collected from each patient at two time points: at admission and on day 7 after admission. This timing aligns with evidence that high-risk COVID-19 patients typically develop severe respiratory distress requiring intensive care around day 7, while others remain stable or improve [23,24,25,26]. Healthy, age- and sex-matched controls (Table S1) were recruited to establish baseline biological reference values for ELISA. Healthy controls and sex-matched COVID-19 patients were of similar age. Control inclusion criteria were age over 18 years and a negative SARS-CoV-2 PCR test at enrollment. Exclusion criteria comprised acute or chronic inflammatory conditions, autoimmune diseases, and the use of immunomodulatory medications.

During venipuncture, blood was collected into citrate tubes for coagulation testing (D-dimer and fibrinogen) and cytokine analysis (IL-17 and IL-10). All samples were processed within 45 min of collection. Serum tubes were used for ferritin, LDH, CRP, and IL-6 analysis, while EDTA tubes were collected for complete blood counts. These tubes were allowed to clot before centrifugation at 1430× *g* for 10 min; the separated serum was then either analyzed immediately for biochemical markers or assayed for IL-6. Citrate tubes were centrifuged at 1230× *g* for 20 min to separate plasma. Plasma samples were either placed directly on the analyzer for D-dimer and fibrinogen testing, or aliquoted and stored at −80 °C (maximum 6 months) for future IL-17 and IL-10 analysis. Neutrophil and lymphocyte counts were measured immediately from EDTA whole blood.

#### 2.2.2. Determination of Acute-Phase Proteins

CRP levels were quantified using a particle-enhanced turbidimetric immunoassay (CRP Extended Range [RCRP] Flex^®^, Siemens Healthcare Diagnostics Inc, Tarrytown, NY, USA). The assay relies on anti-CRP antibody-coated synthetic particles that aggregate in the presence of CRP, creating a measurable turbidity. Calibration used five duplicate-measured calibrators and was repeated every 60 days per reagent lot.

Ferritin levels were measured using the IMMULITE^®^ 2000 solid-phase (Siemens Healthcare Diagnostics Inc, Tarrytown, NY, USA), two-site chemiluminescent immunometric assay (30-min incubation), with two-point calibration (low/high) performed every four weeks.

Fibrinogen was measured using a modified Clauss method [27], determining clotting time in citrated plasma after adding excess thrombin (Multifibren^®^ U reagent, Siemens Healthineers, Erlangen, Germany). The calibration curve was established using standard fibrinogen calibrators analyzed on the coagulation analyzer.

D-dimer levels were determined photometrically using the STA^®^-Liatest^®^ D-Di assay (Diagnostica Stago S.A.S, Asnières-sur-Seine, France). Briefly, latex microparticles coated with D-dimer-specific monoclonal antibodies were mixed with the plasma sample. Antigen-antibody reactions caused microparticle agglutination, increasing the turbidity of the suspension. Kit reagents were pre-calibrated, with the calibration valid for all kits within the same lot.

LDH activity was determined colorimetrically by measuring the NAD+-dependent oxidation of L-lactate to pyruvate at pH 9.4. The resulting formation of NADH was monitored kinetically at 340/700 nm (LDH Flex^®^, Siemens Healthcare Diagnostics, Marburg, Germany), where the rate of absorbance change is directly proportional to the enzyme concentration. A three-level calibrator was measured in triplicate to establish the calibration curve. The calibration interval was 90 days, as recommended.

#### 2.2.3. Determination of Immune Cell-Based Blood Inflammatory Indices

Complete blood cell counts and detailed leukocyte differentials were generated using advanced fluorescence-based flow cytometry. This method combines physical light scatter (resolving cell size and internal complexity) with fluorescent nucleic acid tagging. These joint measurements of granularity and fluorescence intensity isolate distinct populations of monocytes, lymphocytes, neutrophils, eosinophils, and basophils. The neutrophil-to-lymphocyte ratio (NLR), calculated as the absolute neutrophil count divided by the absolute lymphocyte count, was included as a covariate in the statistical analyses.

Blood IL-6 levels were measured using a solid-phase, enzyme-labeled, chemiluminescent sequential immunometric assay with two 30-min incubation cycles (IMMULITE 2000 IL-6, Siemens Healthineers, Forchheim, Germany). The assay was calibrated biweekly using high- and low-concentration calibrators, yielding an analytical sensitivity of 2 pg/mL.

Reference ranges for CRP, ferritin, fibrinogen, D-dimer, LDH, neutrophil and lymphocyte counts, and IL-6 were based on manufacturer-provided values and were verified in a local sample of 10 healthy subjects to confirm their applicability to the study population and equipment (Table S1). All measurements were performed in singular, standardized conditions within the same laboratory. To ensure quality control of the analytical process, including reagent monitoring and analyzer performance verification, two levels of the manufacturer’s control material were analyzed twice daily.

We quantified IL-17 and IL-10 concentrations using solid-phase sandwich ELISA kits (DuoSet, R&D Systems, Minneapolis, MN, USA) and measured absorbance at 450 nm on a Multiskan FC microplate reader (Thermo Fisher Scientific Oy, Vantaa, Finland). All measurements were running in duplicate. Inter-assay reproducibility was monitored using sample pools with high cytokine concentrations. The internal standards, calibrated against purified recombinant human proteins, served as daily calibration curves. The minimum detectable dose (MDD) was defined as the mean optical density of 20 zero-replicates plus two standard deviations. The MDD was 8.05 pg/mL for IL-17 and 11.5 pg/mL for IL-10. Neither cytokine was detectable in healthy control plasma.

### 2.3. Statistical Analysis

To explore sex differences in predicting COVID-19 outcomes, data were stratified by sex. Categorical variables were presented as counts and percentages, and group differences were analyzed using the Pearson Chi-square test or Fisher’s exact test, as appropriate. Data distribution was assessed using the Shapiro–Wilk test. Continuous variables with a normal distribution were reported as mean ± standard deviation (SD). Group differences were evaluated using the independent samples t-test for two-group comparisons, or a one-way analysis of variance (ANOVA) followed by Tukey’s post hoc test for comparisons involving more than two groups. Non-normally distributed variables were presented as median and interquartile range (IQR). Data were analyzed using the Mann–Whitney U test for two-group comparisons, or the Kruskal–Wallis test followed by Dunn’s post hoc test for multiple-group comparisons. Blood indices that differed significantly between severity groups were evaluated using univariate logistic regression. This assessed the relationship between independent variables (acute-phase proteins and immune cell-related indices) and dependent variables (COVID-19 severity or mortality). Results were reported as odds ratios (OR) and 95% confidence intervals (CI). 

Variables with a *p*-value less than 0.10 in the univariate analysis were included in the multivariable logistic regression. Although the 10 events-per-variable (EPV) ratio is a traditional rule of thumb [28], modern methodological literature indicates it is not an absolute threshold; an EPV of 5 to 9 is acceptable depending on the clinical context and does not compromise model validity [29]. We performed rigorous post-estimation diagnostics to justify the inclusion of all selected predictors. First, we assessed multicollinearity using the variance inflation factor (VIF), considering values > 5 as evidence of significant collinearity. Second, we verified numerical stability by ensuring standard errors (SE) remained below <2.0. Third, we evaluated model calibration via the Hosmer–Lemeshow goodness-of-fit test, where a *p*-value > 0.05 indicated adequate fit. Finally, we calculated the Nagelkerke R2 to estimate the proportion of explained variance, where values > 0.20 were acceptable. To ensure robust findings, we excluded models displaying instability or significant multicollinearity.

ROC curves evaluated predictive efficacy and optimal cut-off values for individual and combined model predictors. Because a predictor’s performance can change when integrated into a model, model predictors were re-evaluated in subsequent ROC analyses. These analyses focused on sub-cohorts exceeding the optimal cut-off for each predictor, following previously described methods [30].

The area under the curve (AUC) was used to measure overall diagnostic accuracy from 0.5 to 1.0: <0.5 AUC: no discrimination; ≤0.5 AUC < 0.6: failed/worthless discrimination; ≤0.6 AUC < 0.7: poor/weak discrimination; ≤0.7 AUC < 0.8: fair/acceptable discrimination; ≤0.8 AUC < 0.9: good/clinically useful discrimination, and ≤0.9 AUC < 1.0: excellent/outstanding discrimination [31].

The optimal cut-off points were determined using the Youden index to maximize classification accuracy. Youden index was calculated based on the sensitivities and specificities for all possible cut-off values of a diagnostic test and the cut-off value that yielded the maximum Youden index value (closest to 1) was then identified as the optimal threshold. Statistical analyses were performed using IBM SPSS Statistics, version 23.0 (IBM Corp, Armonk, NY, USA) and GraphPad Prism, version 8.0 (GraphPad Software, San Diego, CA, USA). A two-sided *p*-value ≤ 0.05 was considered statistically significant.

## 3. Results

### 3.1. Baseline Demographic and Clinical Characteristics of COVID-19 Subjects at Admission

At admission, 46% of males and 28% of females exhibited severe-to-critical illness. Males and females with mild-to-moderate COVID-19 shared similar age distributions (Table 1). Female patients with severe-to-critical COVID-19 trended older than male patients, but this variation did not reach statistical significance (Table 1). Additionally, females with severe-to-critical COVID-19 were (*p* = 0.001) older than those with mild-to-moderate disease, a pattern not observed in males (Table 1).

Baseline comorbidities (including cardiovascular disease (CVD) diabetes (DM), and cancer) did not differ significantly between groups of similar disease severity (Table 1). Additionally, CVD was more prevalent in severely-to-critically ill patients (*p* = 0.026 and *p* < 0.001 in males and females, respectively) than in mild-to-moderate cases (Table 1). Furthermore, unlike the mild-to-moderate group (where no patients had DM) a few male and female patients with severe-to-critical disease did suffer from DM (Table 1).

At admission, severely-to-critically ill patients of both sexes exhibited higher (*p* < 0.001) chest CT scores compared with sex-matched subjects with mild-to-moderate disease, indicating more extensive and severe COVID-19 pneumonia (Table 1).

Male (*p* < 0.001) and female (*p* = 0.003) COVID-19 subjects with severe-to-critical disease required more oxygen supplementation compared with sex-matched subjects with mild-to-moderate disease (Table 1). There was no statistically significant difference in oxygen supplementation levels between male and female patients, irrespective of disease severity (Table 1). Additionally, among severely-to-critically ill patients, a significant proportion of males (*p* = 0.006) and an even higher (*p* < 0.001) percentage of females required intubation (Table 1).

Among severely-to-critically ill patients, males experienced lower mortality than females (Table 1). No deaths occurred among patients with mild-to-moderate disease (Table 1).

Co-medication analysis revealed that, irrespective of sex, the use of corticosteroids and aspirin was significantly higher (*p* < 0.001) in severely-to-critically ill patients than in those with mild-to-moderate disease (Table 1). Corticosteroid and aspirin use did not differ by sex among patients with similar disease severity (Table 1). Favipiravir, an antiviral medication, was administered exclusively to subjects with mild/moderate disease, and proportions of males and females receiving this medication were comparable (Table 1).

### 3.2. Sex-Specific Capacity of Inflammatory-Immune Blood Indices to Predict Severe COVID-19

#### 3.2.1. Acute-Phase Proteins as Predictors of Severe COVID-19

To identify acute-phase proteins linked to COVID-19 progression, their values in mild-to-moderate and severe-to-critical patients were compared using purposeful univariate selection, multivariable logistic regression, and ROC curve analysis.

##### At Admission

In healthy males and females, the blood levels of acute-phase proteins were within laboratory reference ranges (Table S1). Among these subjects, only ferritin levels showed a statistically significant sex-related difference, being lower (*p* = 0.032) in females than in males (Table S1).

At admission, CRP, ferritin, D-dimer, and LDH levels were significantly elevated in severe-to-critical COVID-19 patients compared to sex-matched mild-to-moderate cases (Table 2, Figure S1a). Conversely, elevated fibrinogen levels (*p* = 0.041) uniquely characterized females with severe-to-critical illness relative to their mildly-to-moderately ill counterparts (Table 2, Figure S1a).

Univariate analysis showed that elevated CRP (*p* = 0.042 and *p* = 0.002 in males and females, respectively), ferritin (*p* < 0.001 and *p* = 0.002 in males and females, respectively), D-dimer (*p* = 0.003 and *p* = 0.014 in males and females, respectively), and LDH (*p* = 0.002 and *p* = 0.001) in males and females, respectively, levels were associated with severe-to-critical COVID-19 in both males and females. Notably, elevated fibrinogen was associated with severity only in females (*p* = 0.013) (Table 2).

Next, a multivariable logistic regression was performed to estimate independent effects (Table 2). The analysis revealed that elevated ferritin (*p* = 0.011 and *p* = 0.01 for males and females, respectively) and D-dimer levels (*p* = 0.034 and *p* = 0.013 for males and females, respectively) predicted progression to severe-to-critical disease in both males and females (Table 2).

**Figure 2:**
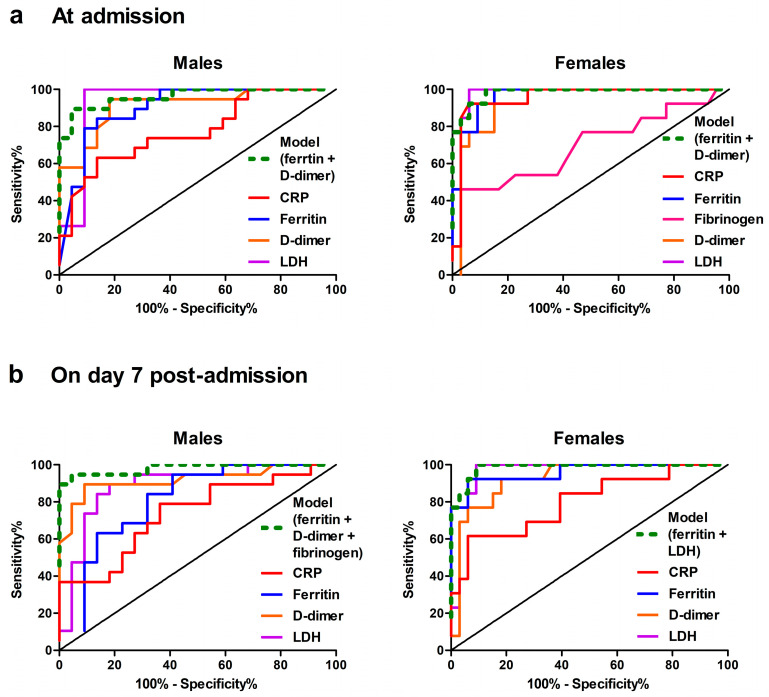
**Sex-specific prediction of COVID-19 severity using acute-phase proteins at admission and on day 7 after admission.** Receiver operating characteristic (ROC) curve analysis was performed (**Panel a**) at admission and (**Panel b**) on day 7 after admission. Solid lines denote the predictive accuracy of individual predictors. (**Panel a**) shows the performance of CRP, ferritin, D-dimer, and LDH in males (**left**) and CRP, ferritin, D-dimer, LDH, and fibrinogen in females (**right**). (**Panel b**) shows CRP, ferritin, D-dimer, and LDH in males (**left**) and females (**right**). Dashed lines show the predictive accuracy of combined models. (**a**) Ferritin + D-dimer in males (**left**) and females (**right**). (**b**) Ferritin + D-dimer + fibrinogen in males (**left**), and ferritin + LDH in females (**right**). The diagonal black line represents a test with no predictive power (AUC < 0.5). The *y*-axis represents sensitivity% (true positive rate) and the *x*-axis represents 100%-specificity% (false positive rate). Area under the curve (AUC) values (quantitative measure of prognostic accuracy), sensitivity, specificity, cut-off values for markers and model are displayed in Table 3. Abbreviations: CRP, C-reactive protein; LDH, lactate dehydrogenase.

To evaluate how effectively the predictor variables and model discriminated between positive and negative COVID-19 cases, ROC analysis was performed. Based on AUC, almost all acute-phase proteins demonstrated outstanding prognostic accuracy (AUC > 0.900) (Table 3, Figure 2a). The only exceptions were CRP in males (AUC: 0.774, 95%CI: 0.630–0.918) and fibrinogen in females (AUC: 0.696, 95%CI: 0.504–0.887) (Table 3, Figure 2a).

ROC analysis showed that optimal early cut-off values differed by sex for all shared variables. This sex discrepancy was particularly striking for ferritin (929.00 ng/mL in males vs. 191.00 ng/mL in females) and D-dimer (0.505 mg/L in males vs. 0.840 mg/L in females) (Table 3, Figure 2a).

ROC analysis also assessed the predictive capacity of the combined ferritin and D-dimer model. For both male and female COVID-19 patients, this combination yielded a higher AUC and a narrower 95% CI than either predictor alone (Table 3, Figure 2a). At the optimal cut-off, the model demonstrated sex-specific performance. Sensitivity (the true positive rate) was lower in males, whereas specificity (the true negative rate) was higher.

Given that individual cut-offs may not be optimal within complex multivariable models, the joint D-dimer-ferritin model was further evaluated for predicting disease progression using sequential ROC analyses [30]. These analyses tested the predictive capacity of each biomarker (variable) within sub-cohorts of patients with supra cut-off levels of the other variable [30]. The prognostic accuracy of D-dimer (AUC) remained significant in both male (*p* = 0.014) and female (*p* = 0.012) sub-cohorts with supra-optimal ferritin cut-offs (Table 3 and Table S2). However, compared to the sex-matched entire cohort, the optimal D-dimer cut-offs increased to 0.84 mg/L in males and 1.40 mg/L in females. This indicates a subgroup where the combined presence of both factors exceeds a risk threshold that neither might reach alone (Table 3 and Table S2). Similarly, ferritin retained its prognostic accuracy in both male (*p* = 0.05) and female (*p* = 0.009) sub-cohorts of COVID-19 patients with supra-optimal D-dimer cut-off values (Table 3 and Table S2). However, the optimal ferritin cut-off value was higher (395.50 ng/mL) in the sub-cohort than in the entire cohort only among female patients (Table 3 and Table S2).

##### On Day 7 After Admission

On day 7 after admission, both male and female severally/critically ill COVID-19 patients exhibited higher blood levels of acute-phase proteins except for fibrinogen when compared with sex-matched patients with mild-to-moderate disease (Table 2, Figure S1b). Although blood levels of fibrinogen were decreased in patients with severe-to-critical disease regardless of sex, the difference reached statistical significance (*p* = 0.036) only in males (Table 2, Figure S1b).

Next, the association between COVID-19 severity and acute-phase protein blood levels was reassessed. Univariate logistic regression analysis revealed that elevated blood levels of CRP (*p* = 0.024 and *p* = 0.01 in males and females, respectively), ferritin (*p* = 0.004 and *p* = 0.005 in males and females, respectively), D-dimer (*p* = 0.008 and *p* = 0.019 in males and females, respectively) and LDH (*p* = 0.002 and *p* = 0.001 in males and females, respectively) were associated with severe-to-critical disease in males and females (Table 2).

Multivariable logistic regression identified elevated ferritin, elevated D-dimer, and decreased fibrinogen as independent predictors of severe or critical disease in male patients. Consequently, these three variables were included in the predictive model for adverse outcomes (Table 2). For female patients, the initial multivariable model encompassed elevated blood levels of ferritin, D-dimer, and LDH (Table 2); however, only ferritin emerged as an independent predictor of severe-to-critical COVID-19 (*p* = 0.045). To optimize model stability and assess individual variable contributions, the Nagelkerke R^2^ of the full model was compared against reduced versions. Stepwise elimination led to the exclusion of D-dimer (OR: 0.897, 95%CI: 0.788–1.020, *p* > 0.05). Conversely, LDH was retained alongside ferritin because its inclusion significantly enhanced the model’s overall stability and predictive accuracy.

To assess the predictive capacity of the acute-phase proteins, ROC analysis was performed. Considering the optimal cut-off for AUC clinical utility, in males all acute-phase proteins, except for CRP (AUC: 0.737, 95%CI: 0.582–0.891), were of clinical utility in predicting progression to severe-to-critical COVID-19, but only the increased blood level of D-dimer exhibited outstanding predictive capacity (Table 3, Figure 2b). Differently, in females, all predictors exhibited outstanding predictive performances except for CRP blood level (AUC: 0.809, 95%CI: 0.664–0.953) (Table 3, Figure 2b). ROC curve analysis also identified sex-specific cut-offs for predicting severe-to-critical COVID-19. These differences were most pronounced for ferritin (males: 575.00 ng/mL vs. females: 237.50 ng/mL) and CRP (males: 6.95 mg/L vs. females: 21.80 mg/L) (Table 3).

ROC analysis also evaluated the predictive capacity of the combined models in males and females. The combined models yielded higher AUC values and narrower 95% CIs than any single predictor (Table 3, Figure 2b). Finally, the multivariable models predicting progression to severe-to-critical COVID-19 in males were subjected to an in-depth evaluation of predictive performance using sequential ROC analyses of: (i) ferritin and fibrinogen in the sub-cohort with supra-optimal cut-off blood levels of D-dimer, (ii) D-dimer and fibrinogen in the sub-cohort with supra-optimal cut-off values of ferritin, and (iii) D-dimer and ferritin in the sub-cohort with sub-optimal cut-off values of fibrinogen [30]. In the sub-cohort with supra-optimal D-dimer values, neither ferritin nor fibrinogen retained statistically significant predictive accuracy (Table S3). This lack of significance may indicate conditional independence or saturated risk. Specifically, once D-dimer reaches the threshold, the additional prognostic information provided by ferritin or fibrinogen becomes negligible or statistically non-discernible within this subgroup. In the sub-cohort with supra-optimal values of D-dimer, neither ferritin nor fibrinogen retained statistically significant predictive accuracy (Table S3). Clinically, although significant for the entire male cohort, these predictors were insignificant in the high-risk sub-cohort, suggesting limited clinical utility for predicting extreme-risk cases. In the male sub-cohort with supra-optimal ferritin levels, both D-dimer (*p* = 0.002) and fibrinogen (*p* < 0.001) retained predictive accuracy (Table S3). Notably, the optimal cut-off for D-dimer was 1.140 mg/L (higher than in the entire cohort), whereas the optimal cut-off for fibrinogen was 3.20 g/L (Table 3 and Table S3). Sequential ROC analysis in males also showed that D-dimer and ferritin maintained predictive accuracy in the sub-cohort with sub-optimal fibrinogen values (Table 3 and Table S3). In this sub-cohort, the optimal cut-off for D-dimer (0.375 mg/L) was lower than in the entire cohort, while the ferritin cut-off (571.00 ng/mL) remained identical (Table 3 and Table S3).

#### 3.2.2. Evaluation of Blood Immune Cell-Related Indices as Predictors of Severe-to-Critical Outcomes

##### At Admission

At admission, blood counts of neutrophils and lymphocytes, the NLR, and pro-inflammatory (IL-6 and IL-17) and anti-inflammatory (IL-10) were also measured. Notably, none of these indices exceeded the laboratory reference ranges in healthy males or females (Table S1).

At admission, severe-to-critical COVID-19 patients of both sexes exhibited higher (*p* < 0.0001) neutrophil counts and NLR, but lower (*p* = 0.003 and *p* < 0.0001 in males and females, respectively) lymphocyte counts, compared to sex-matched mild-to-moderate cases (Table 4, Figure S2a). Unlike IL-6 and IL-10, which were detectable in most COVID-19 patients, IL-17 blood levels were detectable in fewer than 50% of patients (Table S4). Consequently, IL-17 was excluded from further analysis to ensure reliability of the study’s conclusions [32]. The blood level of IL-6 was higher in severely/critically ill male and female patients than in sex-matched mild-to-moderate cases, but this difference reached statistical significance (*p* = 0.016) only in females (Table 4, Figure S2a). However, no statistically significant differences were observed in IL-10 blood levels between patients with mild-to-moderate and severe-to-critical COVID-19 in either males or females (Figure S2a).

Next, the predictive capacity of immune cell-related inflammatory blood indices that significantly differed between severity groups was evaluated using univariate regression analysis. In males, elevated neutrophil counts (*p* = 0.002), an increased NLR (*p* = 0.003), and decreased lymphocyte counts (*p* = 0.006) were associated with severe-to-critical COVID-19. (Table 4). In female COVID-19 patients, only an elevated neutrophil count (*p* = 0.002) and decreased lymphocyte count (*p* = 0.002) correlated with severe-to-critical disease (Table 4).

Subsequent multivariable regression analysis revealed that the combination of elevated neutrophil count and decreased lymphocyte count was an effective predictor of severe-to-critical COVID-19 among males (Table 4). Unlike in males, no predictive model could be generated for females, potentially due to the small size of the female COVID-19 cohort.

**Figure 3:**
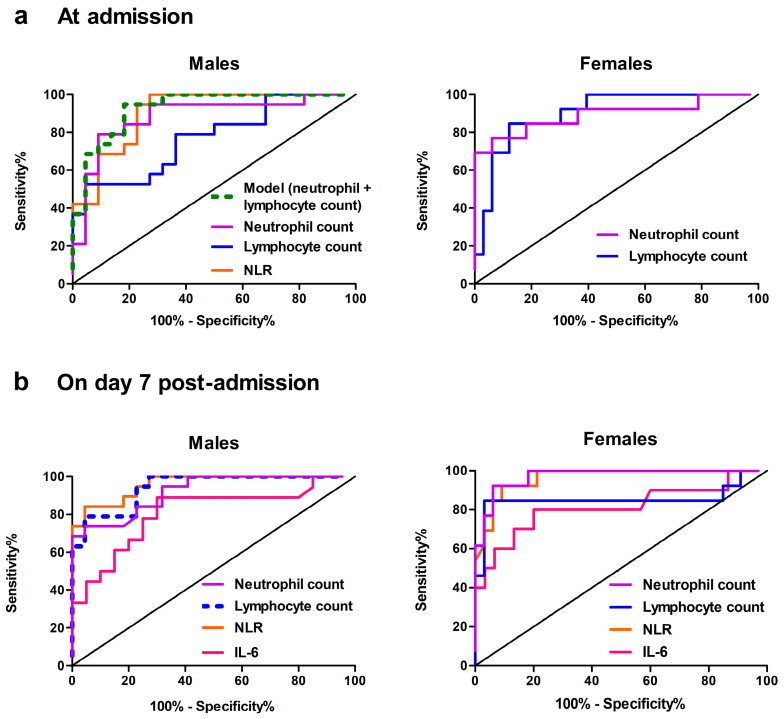
**Sex-specific prediction of COVID-19 severity using immune cell-related indices at admission and on day 7 after admission.** Receiver operating characteristic (ROC) curve analysis was performed (**Panel a**) at admission and (**Panel b**) on day 7 after admission. Solid lines denote the predictive accuracy of individual predictors. (**Panel a**) shows neutrophil counts, lymphocytes count, and NLR in males (**left**) and neutrophil counts and lymphocyte counts in females (**right**). (**Panel b**) shows neutrophil counts, lymphocyte counts, NLR, and IL-6 in males (**left**) and females (**right**). Dashed lines indicate the predictive accuracy of (**Panel a**) combined model (neutrophil counts + lymphocyte counts) in males (**left**) and (**Panel b**) independent predictor (lymphocyte count) in males (**left**). Diagonal black line represents a test with no diagnostic ability (AUC < 0.5). *y*-axis represents sensitivity% (true positive rate) and *x*-axis represents 100%-specificity% (false positive rate). Area under the curve (AUC) values (quantitative measure of predictive accuracy), sensitivity, specificity and cut-off values for immune cell-related indices and model are displayed in Table 5. Abbreviations: NLR, neutrophil-to-lymphocyte ratio; IL, interleukin.

ROC analyses evaluating the discriminative capacity of predictors in males with COVID-19 revealed clinically significant AUC values for neutrophil count and NLR (Table 5, Figure 3a). Conversely, the AUC for lymphocyte count was below 0.80, indicating limited clinical utility (Table 5, Figure 3a). The analysis also showed clinical utility of individual neutrophil and lymphocyte counts in predicting progression to severe-to-critical outcomes in females (Table 5, Figure 3a).

The ROC analysis also revealed that the combined model (neutrophil and lymphocyte counts) yielded a higher AUC and a narrower 95% CI in males compared to any single predictor (Table 5, Figure 3a). The optimal cut-off for lymphocyte count did not differ between males and females, whereas that for neutrophil count (5.67 × 10^9^/L in males vs. 4.96 × 10^9^/L in females) was higher in males than in females (Table 5).

Next, sequential ROC analysis evaluated the male-specific multivariable model for severe disease. This analysis assessed neutrophil and lymphocyte performance in sub-groups where neutrophil count and lymphocyte count were above and below optimal cut-off values, respectively. Neither neutrophil counts in the low-lymphocyte sub-cohort nor lymphocyte counts in the high-neutrophil sub-cohort achieved statistically significant prognostic accuracy (Table S5). This indicates that both variables were strong predictors. Combined, these strong predictors offered a holistic view of disease outcome drivers.

##### On Day 7 After Admission

On day 7 after admission, severely/critically ill COVID-19 patient exhibited a lower lymphocyte count (*p* < 0.0001 and *p* = 0.0008 in males and females, respectively), a higher neutrophil count (*p* < 0.0001), and a higher NLR (*p* < 0.0001) than sex-matched mild-to-moderate cases (Table 4, Figure S2b). Consistent with the first assessment, only IL-6 and IL-10 were included in subsequent statistical analyses (Table S4). Blood IL-6 levels were higher (*p* = 0.0013 and *p* = 0.0037 in males and females, respectively) in both males and females with severe-to-critical disease compared to sex-matched patients with mild-to-moderate disease. No statistically significant differences were observed for the blood level of IL-10 between patients with mild-to-moderate disease and patients with severe-to-critical disease (Figure S2b).

Univariate regression analysis linked severe-to-critical COVID-19 to elevated neutrophil counts (*p* = 0.001 and *p* = 0.003 in males and females, respectively), increased NLR (*p* = 0.006 and *p* = 0.001 in males and females, respectively), and decreased lymphocyte counts (*p* = 0.002 and *p* = 0.0008 in males and females, respectively) in both sexes (Table 4). Furthermore, this severe disease state was associated with elevated IL-6 blood levels (*p* 0 0.049 and *p* = 0.047 in males and females, respectively) across both sexes (Table 4).

Subsequent multivariable regression analysis revealed that, after controlling for other factors in male COVID-19 subjects, lymphopenia was the only variable significantly (*p* = 0.008) associated with severe-to-critical disease in males (Table 4).

ROC analysis showed that all predictors of severe-to-critical COVID-19 had clinically useful AUC values in males and females (Table 5, Figure 3b). At this evaluation point, optimal cut-offs for neutrophil count (9.85 × 10^9^/L in males vs. 6.62 × 10^9^/L in females) and NLR (6.66 in males vs. 3.52 in females) were significantly higher in males than in females (Table 5). This finding underscores the necessity of sex-based evaluation when assessing subjects with COVID-19.

### 3.3. Acute-Phase Proteins and Immune Cell-Related Indices for Mortality Prediction in COVID-19

Next, the association of inflammatory-immune blood indices with COVID-19 mortality was assessed in severely-to-critically ill patients.

#### 3.3.1. Acute-Phase Proteins as Predictors

##### At Admission

At admission, severely-to-critically ill male COVID-19 non-survivors exhibited significantly higher blood LDH (*p* = 0.035) and females D-dimer levels (*p* = 0.0186) than survivors among both males and females (Figure S3a). None of these acute-phase proteins significantly correlated with COVID-19 mortality in either sex.

##### On Day 7 After Admission

On day 7 after admission, severely-to-critically female ill non-survivors exhibited higher (*p* = 0.006) blood LDH and D-dimer levels than sex-matched survivors (Table 6, Figure S3b). Furthermore, among severely-to-critically ill male patients, CRP levels were higher (*p* = 0.007) in male non-survivors compared to their respective surviving counterparts (Table 6, Figure S3b).

Univariate regression analysis revealed that CRP (*p* = 0.027) was associated with mortality in males, whereas no variables predicted death in severely or critically ill females (Table 6).

**Figure 4:**
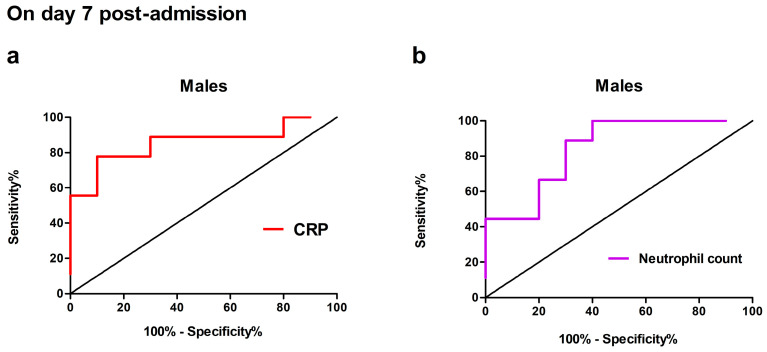
Prediction of death outcome using CRP and neutrophil count in severe-to-critical male COVID-19 patients on day 7 after admission. Solid lines denote the predictive accuracy of individual predictors. (**a**) CRP and (**b**) neutrophil count in males. Diagonal black line represents a test with no diagnostic ability (AUC < 0.5). *y*-axis represents sensitivity% (true positive rate) and *x*-axis represents 100%-specificity% (false positive rate). Area under the curve (AUC) values (quantitative measure of diagnostic accuracy), sensitivity, specificity, cut-off values for CRP, and neutrophil count are displayed in Table 7. Abbreviations: CRP, C-reactive protein.

Subsequent ROC analysis identified CRP as a strong, clinically relevant predictor of mortality (AUC: 0.856, 95% CI: 0.670–1.000) among severely-to critically ill male COVID-19 patients (Table 7, Figure 4).

#### 3.3.2. Blood Immune Cell-Related Indices as Predictors

##### At Admission

Among severely-to-critically ill COVID-19 patients, no statistically significant difference was observed in neutrophil counts, lymphocyte counts, or NLR between survivors and non-survivors, regardless of sex (Figure S4a).

Considering the percentage of cases with detectable cytokine levels, only blood IL-6 and IL-10 cytokine levels were statistically evaluated (Table S6). Cytokine evaluation revealed that while both male and female non-survivors had higher IL-6 levels than sex-matched survivors, this difference was only statistically significant (*p* = 0.05) in males (Figure S4a). IL-10 blood levels did not statistically differ between non-survivors and survivors in either in males or females (Figure S4a).

At admission, no blood immune cell-related indices correlate with death in severe-to-critical COVID-19 cases.

##### On Day 7 After Admission

Grouping male and female severely or critically ill patients by survival revealed that on day 7 after admission, non-survivors had elevated neutrophil counts (*p* = 0.01 and *p* = 0.002 in males and females, respectively) and NLR (*p* = 0.03 for both sexes) compared to survivors, whereas the lymphocyte counts were comparable between these two groups (Table 6, Figure S4b). Furthermore, among severely or critically ill patients, a lower proportion of females than males exhibited detectable blood IL-6 levels, irrespective of survival status (Table S6). Statistically significant differences were observed for blood IL-6 levels between severely or critically ill non-survivors and survivors only in males, being higher (*p* = 0.05) in non-survivors than in survivors (Table 6, Figure S4b). However, blood IL-10 levels did not statistically significantly differ between non-survivors and survivors (Figure S4b). Unlike non-survivors, none of the female survivors had detectable blood levels of IL-10 (Table S6; Figure S4b).

Seven days after admission, univariate analysis revealed that only neutrophil count correlated (*p* = 0.038) with mortality in severely or critically ill male COVID-19 patients (Table 6, Figure 4). ROC analysis revealed that the neutrophil count was a clinically useful predictor of mortality in severely or critically ill male COVID-19 patients (AUC: 0.844; 95% CI: 0.668–1.00) (Table 7, Figure 4b). Conversely, none of the blood immune-cell inflammatory indices predicted mortality among severely or critically ill female COVID-19 patients (Table 6).

## 4. Discussion

This longitudinal study highlights acute-phase proteins and immune cell indices as early, sex-specific predictors of COVID-19 severity and mortality. Consequently, the independent biomarkers and predictive models derived from t data offer valuable tools for optimizing clinical risk assessment. Additionally, they contribute to our understanding of how initial pathogenesis differs between males and females.

Sex specificities in predictive capacity of acute-phase proteins

At admission, elevated levels of CRP, ferritin, LDH, and D-dimer predicted COVID-19 progression to severe-to-critical disease. Although these biomarkers were common to both sexes, their predictive accuracy and optimal cut-off values differed between males and females. The former implies distinct clinical relevance; for instance, CRP was clinically relevant only in females. Additionally, while fibrinogen was associated with progression to severe-to-critical COVID-19 in females, this predictor lacked clinical relevance.

On day 7 after admission, all acute-phase proteins were associated with severe-to-critical disease progression; however, with distinct criteria (i.e., cut-off levels, sensitivity, and specificity) between sexes. CRP lacked clinical significance in males. These findings are important as despite sex-specific levels of acute-phase proteins being reported, and their links to disease progression being documented in general cohorts [33], data remain insufficient regarding how sex modulates the relationship between these biomarkers and COVID-19 severity.

At admission, ferritin and D-dimer independently predicted progression to severe or critical COVID-19 in both sexes better than any single predictor. Notably, predictive thresholds for severe-to-critical disease differed substantially by sex. Males exhibited strikingly higher ferritin thresholds than females in both the total cohort and the high-D-dimer sub-cohort. Conversely, male D-dimer thresholds were markedly lower in both the total cohort and the high-ferritin sub-cohort. These findings are particularly relevant because (to our knowledge) sex-specific diagnostic thresholds are rarely used in COVID-19 management, and data on variable thresholds within predictive models remain virtually nonexistent. These findings align with data demonstrating that in COVID-19 patients: (i) serum ferritin levels rise significantly with disease severity and are notably higher in males than in females [34,35], and (ii) these levels are not only associated with the progression to severe or critical illness [36,37] but also act as an independent predictor of severity, requiring higher cut-off thresholds in males [34,35]. Given that blood ferritin levels correlate with severity of inflammation [38], the sex difference in ferritin levels also aligns with data showing that males develop stronger inflammatory responses than females, even when matched for COVID-19 severity [39]. Moreover, sex-based differences in ferritin levels align with its central role in driving immune dysregulation. High ferritin levels suppress immunity and trigger pro-inflammatory responses. This leads to hypercytokinemia and severe clinical complications [35,36,40].

The positive association between rising blood D-dimer levels and severe-to-critical disease has been already reported [41,42,43]. Although D-dimer is a standard marker for coagulation activation [44], its early elevation in COVID-19 patients often stems from alveolar inflammation and cytokine production [44] Thus, at an early stage of pathogenesis, D-dimer acts primarily as an acute-phase reactant [42,45,46]. Supporting this, data show that abnormalities in prothrombin time, partial thromboplastin time, and platelet counts are relatively uncommon in COVID-19 patients at initial presentation [42,45,46]. Although not confirmed in our small cohort, healthy males typically have lower baseline D-dimer levels than females [47,48]. Therefore, we speculate that while males may have a more robust virus-induced D-dimer production in the lungs due to greater inflammation [39], this increase is insufficient to overcome the premorbid sex differences in blood D-dimer levels. Finally, although the multivariable logistic regression models for predicting severe-to-critical COVID-19 progression utilized identical variables for both sexes, their cut-off values and predictive performances differed. Specifically, the male-specific model yielded fewer false positives (higher specificity) but missed more true cases (lower sensitivity). These findings are also clinically valuable for evaluating a patient’s risk of adverse disease progression.

Dynamic changes in acute-phase proteins during COVID-19 [23,49] prompted a re-evaluation of their predictive value. All proteins except fibrinogen are associated with progression in both sexes, but with distinct predictive performances. However, on day 7, multivariable risk models differed significantly by sex: males required ferritin, D-dimer, and fibrinogen as independent predictors, whereas females required only ferritin. Although LDH was not causally linked to cell damage in COVID-19 [50] and therefore not independent in predicting disease severity for either sex, it substantially improved the model’s overall predictive capacity in females. To our knowledge, this is the first study to offer sex-specific models including cut-off values for their composite variables. This is clinically important as these are expected to differ from the cut-offs used when independent variables are analyzed alone. The changes in the influence of sex on the predictive capacity of D-dimer during the course of the disease mirror its nature as dynamic predictor, meaning its capacity to independently forecast adverse COVID-19 progression shifts during the disease trajectory [49]. This pattern points to sex-specific kinetics in how the disease develops [51]. Taken together, these two findings corroborate that a decreased fibrinogen level is an independent predictor of severe-to-critical COVID-19 exclusively in males. First, males generally face a higher risk of severe disease [52,53]. Second, fibrinogen levels often drop due to increased consumption during severe infection progression, specifically in cases of sepsis, septic shock, or disseminated intravascular coagulation [54,55]. These conditions are recognized complications of later-stage COVID-19 [42,45,46].

Furthermore, on day 7 after admission, CRP predicted mortality only in males with severe-to-critical COVID-19. This aligns with data linking higher CRP levels (after adjusting for age and comorbidities) with poorer outcomes in hospitalized male patients compared to females [56]. Because CRP actively drives tissue injury [57], these findings may suggest a sex-specific, pathogenic role for CRP during early disease stages.

Sex specificities in predictive capacity of immune cell-related blood indices

At admission and on day 7 after admission, neutrophilia and lymphopenia in male and female COVID-19 patients were associated with progression to severe-to-critical illness. These findings align with previous whole-cohort studies linking these cell counts to COVID-19 severity [58,59,60]. Additionally, elevated NLR was associated with greater severity of COVID-19 in males and females on admission and on day 7 after admission, respectively. This is in line with findings indicating dynamic nature of NLR as disease severity predictor in cohort of COVID-19 patients not segregated by sex [61,62]. Unlike the assessment at admission, on day 7 after admission IL-6 was a non-independent predictor of severe-to-critical disease in both sexes, though its predictive characteristics differed. It showed higher sensitivity but lower specificity in males than in females. This changing predictive value aligns with findings that IL-6 production fluctuates highly and is inconsistently elevated across severity levels [63,64]. Therefore, these findings require further evaluation in larger cohorts.

Furthermore, neutrophilia and lymphopenia were identified as independent prognostic markers of severe-to-critical COVID-19 in males, but not in females. Supporting these sex differences, studies indicate that while neutrophil counts show a severity-dependent increase in both sexes, their predictive utility is limited in females [39]. This limitation has been ascribed to a more “favorable” female immune profile, characterized by robust anti-inflammatory responses and earlier activation of adaptive immunity, which makes the neutrophil count a less specific predictor of severity in women [39]. The sex-specific predictive capacity of lymphopenia in COVID-19 aligns with previous research indicating that lymphocyte depletion has greater predictive weight in males than in females [65,66,67,68]. This divergence may stem from evidence that: (i) viral fusion and replication are accelerated in males [69], and (ii) the male adaptive immune system is more vulnerable to viral degradation [70,71], whereas females mount more robust adaptive immune responses [72]. Seven days after admission, lymphopenia independently predicted COVID-19 progression exclusively in male patients. Given the limited size of the female cohort, these findings warrant reassessment in a larger population.

Finally, multivariable regression analysis showed that on day 7 after admission, elevated neutrophil counts independently predicted mortality only in severely-to-critically ill male COVID-19 patients. This supports previous findings that neutrophilia is less specific for predicting mortality in females. Correspondingly, baseline neutrophil counts in this study were higher in males than in females; however, this difference did not reach statistical significance, likely due to limited cohort sizes. These observations are consistent with evidence that neutrophils contribute to thrombosis and pulmonary infiltrates, both of which have been frequently detected in post-mortem samples of patients with severe SARS-CoV-2 infection [73,74,75].

In conclusion, this longitudinal study identified sex- and time-specific acute-phase proteins and immune cell-related indices associated with severe-to-critical COVID-19. We established independent predictors and, notably, developed predictive models for disease progression that outperform individual predictors. Additionally, we provide the first known clinical cut-offs for model predictors. Moreover, the study suggests a sex- and time-dependent role for these cells and proteins in COVID-19 pathogenesis. Thus, the study may not only be important for better (time-dependent sex-specific) stratification of COVID-19 patients to optimize therapeutic approach, but it may also provide a solid basis for generating sex-specific therapies.

Strengths and limitations

This study is among a limited number of studies investigating how sex affects the predictive strength of routine blood inflammatory indices, including their interactions at different time points during early COVID-19 progression.

However, several limitations must be noted. First, because it was conducted at a single center with a small sample size (especially regarding cytokine data), it should be interpreted as an initial, exploratory phase study. Secondly, excluding patients who died or were discharged before day 7 after admission introduces potential selection bias. This exclusion was necessary to ensure complete longitudinal data, but it may have omitted the most critical or mildest cases. Thirdly, baseline, premorbid values for the examined parameters were unavailable.

## Figures and Tables

**Table 1:** Demographic and clinical characteristics of COVID-19 subjects.

	Mild/Moderate (*n* = 55)		Severe/Critical (*n* = 32)		
Parameter	Males (*n* = 22)	Females (*n* = 33)	Males (*n* = 19)	Females (*n* = 13)	*p*
Age, years	47.36 ± 13.89	49.24 ± 12.88	55.37 ± 16.84	64.46 ± 11.49 ^b^ *p* = 0.001	0.003
***Comorbidities, n* (%)**					
CVD	2 (9.09%)	1 (3.03%)	8 (42.11%) ^a^ *p* = 0.026	9 (69.23%) ^b^ *p* < 0.001	<0.001
DM	0	0	3 (15.79%)	2 (15.38%)	0.028
Cancer	0	0	0	1 (7.69%)	0.124
***COVID-19 severity sign, management*** ***and outcome***					
CT severity score	7.5 (5–10)	6 (4–9)	20 (18–22) ^a^ *p* < 0.001	21 (18–22) ^b^ *p* < 0.001	<0.001
Oxygen supplementation (L/min)	7.5 (4.50–15)	5 (3–6)	30 (18.75–58.75) ^a^ *p* < 0.001	32.5 (18.75–35) ^b^ *p* = 0.003	<0.001
Intubation, *n* (%)	0	0	6 (31.58%) ^a^ *p* = 0.006	7 (53.85%) ^b^ *p* < 0.001	<0.001
Death, *n* (%)	0	0	9 (47.37%) ^a^ *p* < 0.001	8 (61.54%) ^b^ *p* < 0.001	<0.001
***Therapy, n* (%)**					
Corticosteroids	2 (9.09%)	2 (6.06%)	19 (100%) ^a^ *p* < 0.001	13 (100%) ^b^ *p* < 0.001	<0.001
Aspirin	8 (36.36%)	12 (54.55%)	19 (100%) ^a^ *p* < 0.001	13 (100%) ^b^ *p* < 0.001	<0.001
Favipiravir	5 (22.73%)	8 (24.24%)	0	0	0.113
Fraxiparine	16 (72.73%)	22 (66.67%)	14 (73.68%)	11 (84.62%)	0.878
Antibiotics	22 (100%)	33 (100%)	19 (100%)	13 (100%)	NA

COVID-19 patients were classified by disease severity based on the criteria in the Materials and Methods section 2. Categorical variables are presented as absolute frequencies (percentages). Continuous variables are presented as mean ± standard deviation (SD) or median (interquartile range, IQR). ^a^ compared to Mild/Moderate males. ^b^ compared to Mild/Moderate females. *p* ≤ 0.05 was considered statistically significant. Abbreviations: Mild/Moderate, mild-to-moderate illness; Severe/Critical, severe-to-critical illness; CVD, cardiovascular diseases; DM, diabetes mellitus; CT, computed tomography; NA, not applicable.

**Table 2:** Acute-phase proteins as sex-specific predictors of severe COVID-19 at admission and on day 7 after admission: univariate and multivariable logistic regression analysis.

	Males				Females			
	COVID-19 Severity		Univariate Logistic Regression	Multivariable Logistic Regression	COVID-19 Severity		Univariate Logistic Regression	Multivariable Logistic Regression
**Acute-phase** **proteins**	Median (IQR)		OR (95% CI)	OR (95% CI)	Median (IQR)		OR (95% CI)	OR (95% CI)
***At*** ***admission***	*Mild/* *Moderate*	*Severe/* *Critical*			*Mild/* *Moderate*	*Severe/* *Critical*		
CRP (mg/L)	23.05 (4.95–31.50)	52.50 (20.20–95.60) *p* = 0.0029	1.284 (1.010–1.634) *p* = 0.042	-	4.40 (2.55–19.85)	98.20 (53.65–171.0) *p* < 0.0001	1.482 (1.153–1.905) *p* = 0.002	-
Ferritin (ng/mL)	350.0 (274.8–758.5)	1454 (1209–1600) *p* < 0.0001	1.038 (1.018–1.030) *p* < 0.001	1.030 (1.007–1.053) *p*= 0.011	91 (40–166)	685.0 (357.5–1291.0) *p* < 0.0001	1.093 (1.034–1.155) *p* = 0.002	1.100 (1.023–1.255) *p* = 0.010
Fibrinogen (g/L)	3.400 (3.175–4.525)	3.8 (3.0–5.6) *p* = 0.3263	-	-	3.5 (2.9–4.2)	4.2 (3.3–5.5) *p* = 0.0414	2.626 (1.224–5.633) *p* = 0.013	-
D-dimer (mg/L)	0.365 (0.260–0.493)	1.23 (0.85–3.68) *p* < 0.0001	1.537 (1.158–2.038) *p* = 0.003	1.392 (1.025–1.891) *p* = 0.034	0.320 (0.265–0.695)	2.16 (1.14–6.63) *p* < 0.0001	1.153 (1.030–1.292) *p* = 0.014	1.136 (1.027–1.255) *p* = 0.013
LDH (IJ/L)	200.5 (185.8–223.3)	560 (408–873) *p* < 0.0001	1.078 (1.028–1.130) *p* = 0.002	-	181.0 (169.0–234.5)	468.0 (409.5–662.5) *p* < 0.0001	1.163 (1.063–1.272) *p* = 0.001	-
***On day 7*** ***after admission***								
CRP (mg/L)	5.35 (2.68–27.70)	30.0 (8.10–76.40) *p* = 0.0100	1.330 (1.039–1.704) *p* = 0.024	-	2.50 (1.40–9.0)	25.60 (4.05–57.10) *p* = 0.0018	2.246 (1.216–4.146) *p* = 0.010	-
Ferritin (ng/mL)	553.5 (307.5–1029)	1100 (792–1389) *p* = 0.0009	1.027 (1.009–1.046) *p* = 0.004	1.047 (1.004–1.091) *p* = 0.033	124.0 (48.5–177.5)	638.0 (353.5–1006) *p* < 0.0001	1.171 (1.048–1.309) *p* = 0.005	1.154 (1.003–1.326) *p* = 0.045
Fibrinogen (g/L)	3.65 (3.08–4.40)	3.1 (2.7–3.5) *p* = 0.0362	0.464 (0.210–1.024) *p* = 0.057	0.095 (0.010–0.867) *p* = 0.037	3.2 (2.6–3.7)	2.6 (1.8–3.6) *p* = 0.1124	-	-
D-dimer (mg/L)	0.370 (0.268–0.545)	3.58 (1.38–19.51) *p* < 0.0001	1.217 (1.052–1.409) *p* = 0.008	1.354 (1.058–1.733) *p* = 0.016	0.35 (0.28–0.65)	2.380 (1.145–4.805) *p* < 0.0001	1.067 (1.010–1.126) *p* = 0.019	-
LDH (IJ/L)	197.5 (177.3–235.3)	569 (308–753) *p* < 0.0001	1.077 (1.030–1.127) *p* = 0.002	-	198.0 (161.0–221.5)	431.0 (297.5–579.5) *p* < 0.0001	1.176 (1.066–1.297) *p* = 0.001	1.121 (0.995–1.263) *p* = 0.060

Only acute-phase proteins with statistically significant differences between severity groups (severe-to-critical vs. mild-to-moderate) by sex were included in the univariate logistic regression. Severe-to-critical COVID-19 outcome was the dependent variable. Variables with *p* < 0.100 in the univariate analysis were entered into the multivariable model; only final model variables are shown. Data are presented as odds ratios (OR) with 95% confidence intervals (CI). ORs for CRP, ferritin, and LDH represent a 10-fold increase in mg/L, ng/mL, and U/L, respectively; the OR for D-dimer represents a 0.1 mg/L increase. *p* ≤ 0.05 indicates statistical significance. Abbreviations: IQR, interquartile range; CRP, C-reactive protein; LDH, lactate dehydrogenase.

**Table 3:** Receiver operating characteristic (ROC) analysis of acute-phase proteins predicting COVID-19 severity by sex: at admission and on day 7 after admission.

	Males				Females			
Acute-Phase Proteins	AUC (95% CI)	Sensitivity (%)	Specificity (%)	Cut-Off	AUC (95% CI)	Sensitivity (%)	Specificity (%)	Cut-Off
***At admission***								
Model (ferritin + D-dimer)	0.962 (0.909–1) *p* < 0.001	89.5	95.5	-	0.984 (0.956–1) *p* < 0.001	100	87.9	-
CRP (mg/L)	0.774 (0.630–0.918) *p* = 0.003	63.2	86.4	38.7	0.955 (0.892–1) *p* < 0.001	92.3	93.9	31.3
Ferritin (ng/mL)	0.903 (0.809–0.997) *p* < 0.001	84.2	86.4	929	0.965 (0.919–1) *p* < 0.001	100	84.8	191
Fibrinogen (g/L)	-	-	-	-	0.696 (0.504–0.887) *p* = 0.040	46.2	100	4.95
D-dimer (mg/L)	0.914 (0.825–1) *p* < 0.001	94.7	81.8	0.505	0.946 (0.885–1) *p* < 0.001	100	84.8	0.84
LDH (IJ/L)	0.933 (0.842–1) *p* < 0.001	100	90.9	271.5	0.970 (0.918–1) *p* < 0.001	100	93.9	316
***On day 7 after admission***								
Model (ferritin + D-dimer + fibrinogen)	0.981 (0.945–1) *p* < 0.001	94.7	95.5	-	-	-	-	-
Model (ferritin + LDH)	-	-	-	-	0.986 (0.962–1) *p* < 0.001	100	90.9	-
CRP (mg/L)	0.737 (0.582–0.891) *p* = 0.01	78.9	63.6	6.95	0.809 (0.664–0.953) *p* = 0.001	61.5	93.9	21.8
Ferritin (ng/mL)	0.804 (0.666–0.942) *p* = 0.001	94.7	59.1	575	0.960 (0.898–1) *p* < 0.001	92.3	93.9	237.5
D-dimer (mg/L)	0.920 (0.828–1) *p* < 0.001	89.5	90.9	0.65	0.924 (0.845–1) *p* = 0.001	92.3	81.8	0.845
LDH (IJ/L)	0.885 (0.775–0.995) *p* < 0.001	89.5	81.8	270.5	0.963 (0.910–1) *p* < 0.001	100	90.9	241

Data are presented as area under the curve (AUC) and 95% confidence interval (CI). *p* ≤ 0.05 was considered statistically significant. The cut-off values were determined as described in the Materials and Methods section 2. Abbreviations: CRP, C-reactive protein; LDH, lactate dehydrogenase.

**Table 4:** Immune cell-related indices as sex-specific predictors of severe COVID-19 at admission and on day 7 after admission: univariate and multivariable logistic regression analysis.

	Males				Females			
	COVID-19 Severity		Univariate Logistic Regression	Multivariable Logistic Regression	COVID-19 Severity		Univariate Logistic Regression	Multivariable Logistic Regression
**Immune cell-related blood indices**	Median (IQR)		OR (95% CI)	OR (95% CI)	Median (IQR)		OR (95% CI)	OR (95% CI)
***At*** ***admission***	*Mild/* *Moderate*	*Severe/* *Critical*			*Mild/* *Moderate*	*Severe/* *Critical*		
Neutrophil count (10^9^/L)	3.525 (3.048–4.375)	9.464 (5.874–11.580) *p* < 0.0001	1.054 (1.019–1.091) *p* = 0.002	1.051 (1.013–1.089) *p* = 0.007	2.530 (1.685–3.463)	7.146 (4.523–11.740) *p* < 0.0001	1.097 (1.036–1.162) *p* = 0.002	-
Lymphocyte count (10^9^/L)	1.270 (0.965–1.573)	0.894 (0.302–1.226) *p* = 0.0033	0.736 (0.592–0.916) *p* = 0.006	0.766 (0.594–0.988) *p* = 0.040	1.380 (1.020–1.905)	0.655 (0.424–0.873) *p* < 0.0001	0.658 (0.507–0.854) *p* = 0.002	-
NLR	2.40 (1.89–4.13)	12.19 (5.75–21.53) *p* < 0.0001	1.033 (1.011–1.056) *p* = 0.003	-	2.08 (1.23–2.93)	10.31 (7.74–15.12) *p* < 0.0001	1.217 (0.988–1.500) *p* = 0.065	-
IL-6 (pg/mL)	9.50 (4.18–18.53)	16.85 (7.43–31.55) *p* = 0.1000	-	-	6.90 (4.98–16.38)	13.60 (10.60–137.00) *p* = 0.0158	1.530 (0.885–2.643) *p* = 0.128	-
***On day 7*** ***after admission***								
Neutrophil count (10^9^/L)	3.145 (2.788–4.690)	12.75 (5.92–17.93) *p* < 0.0001	1.041 (1.017–1.066) *p* = 0.001	-	2.96 (2.40–4.19)	10.52 (7.47–15.30) *p* < 0.0001	1.117 (1.037–1.203) *p* = 0.003	-
Lymphocyte count (10^9^/L)	1.600 (1.318–1.873)	0.717 (0.431–0.873) *p* < 0.0001	0.529 (0.350–0.799) *p* = 0.002	0.456 (0.256–0.815) *p* = 0.008	2.00 (1.44–2.31)	0.806 (0.509–1.146) *p* = 0.0008	0.823 (0.726–0.934) *p* = 0.003	-
NLR	2.15 (1.59–3.54)	19.59 (9.42–28.44) *p* < 0.0001	1.040 (1.011–1.068) *p* = 0.006	-	1.60 (1.26–2.43)	12.67 (7.41–24.97) *p* < 0.0001	1.047 (1.019–1.076) *p* = 0.001	-
IL-6 (pg/mL)	4.75 (2.88–8.23)	16.15 (7.53–62.15) *p* = 0.0013	1.895 (1.003–3.580) *p* = 0.049	-	3.80 (2.78–6.23)	25.30 (5.95–115.4) *p* = 0.0037	1.972 (1.009–3.853) *p* = 0.047	-

Only immune cell-related indices with statistically significant differences between severity groups (severe-to-critical vs. mild-to-moderate) by sex were included in the univariate logistic regression. Severe-to-critical COVID-19 outcome was the dependent variable. Variables with *p* < 0.100 in the univariate analysis were entered into the multivariable model; only final model variables are shown. Data are presented as odds ratios (OR) with 95% confidence intervals (CI). OR for IL-6 was determined for every 10 pg/mL increase. OR for neutrophil and lymphocyte counts were calculated for each 0.1 × 10^9^/L increase and decrease, respectively, while the OR for NLR was based on every 0.1 unit increase in the ratio. *p* ≤ 0.05 indicates statistical significance. Abbreviations: IQR, interquartile range; NLR, neutrophil-to-lymphocyte ratio; IL, interleukin.

**Table 5:** Receiver operating characteristic (ROC) analysis of immune cell-related indices predicting COVID-19 severity by sex: at admission and on day 7 after admission.

	Males				Females			
Immune Cell-Related Blood Indices	AUC (95% CI)	Sensitivity (%)	Specificity (%)	Cut-Off	AUC (95% CI)	Sensitivity (%)	Specificity (%)	Cut-Off
***At*** ***admission***								
Model (neutrophil count + lymphocyte count)	0.928 (0.852–1) *p* < 0.001	94.7	81.8	-	-	-	-	-
Neutrophil count (10^9^/L)	0.883 (0.770–0.994) *p* < 0.001	78.9	90.9	5.67	0.893 (0.767–1) *p* < 0.001	76.9	93.9	4.96
Lymphocyte count (10^9^/L)	0.770 (0.626–0.915) *p* = 0.003	52.6	95.5	0.9	0.902 (0.812–0.993) *p* < 0.001	84.6	87.9	0.9
NLR	0.904 (0.814–0.995) *p* < 0.001	100	72.7	3.463	-	-	-	-
***On day 7*** ***after admission***								
Neutrophil count (10^9^/L)	0.920 (0.841–0.999) *p* < 0.001	73.7	95.5	9.85	0.972 (0.932–1) *p* < 0.001	92.3	93.9	6.62
Lymphocyte count (10^9^/L) ^a^	0.943 (0.879–1) *p* < 0.001	78.9	95.5	1.1	0.823 (0.643–1) *p* = 0.001	84.6	93.9	1.18
NLR	0.959 (0.908–1) *p* < 0.001	84.2	95.5	6.661	0.965 (0.919–1) *p* < 0.001	92.3	90.9	3.52
IL-6 (pg/mL)	0.807 (0.663–0.951) *p* = 0.002	88.9	70.0	5.9	0.812 (0.627–0.996) *p* = 0.003	80	80	6.7

Data are presented as area under the curve (AUC) and 95% confidence interval (CI). *p* ≤ 0.05 was considered statistically significant. ^a^ multivariable regression analysis revealed that, in male COVID-19 subjects, decreased lymphocyte count was the only independent predictor linked with severe-to-critical disease on day 7 after admission. The cut-off values were determined as described in the Materials and Methods section. 2 Abbreviations: NLR, neutrophil-to-lymphocyte ratio; IL, interleukin.

**Table 6:** Acute-phase proteins and neutrophil count as sex-specific predictors of mortality on day 7 after admission: univariate logistic regression analysis.

	Males			Females		
	COVID-19 Outcome		Univariate Logistic Regression	COVID-19 Outcome		Univariate Logistic Regression
**On day 7** **after admission**	Median (IQR)		OR (95% CI)	Median (IQR)		OR (95% CI)
***Acute-phase*** ***proteins***	*Survivors*	*Non-survivors*		*Survivors*	*Non-survivors*	
CRP (mg/L)	9.65 (4.58–30.05)	76.4 (34.5–124.6) *p* = 0.0076	1.612 (1.054–2.465) *p* = 0.027	22.30 (4.05–48.00)	28.95 (3.35–83.35) *p* = 0.7242	-
D-dimer (mg/L)	2.23 (0.63–11.24)	5.66 (2.70–21.61) *p* = 0.2110	-	0.98 (0.69–1.85)	3.82 (2.30–5.99) *p* = 0.0062	1.285 (0.953–1.733) *p* = 0.100
LDH (IJ/L)	371.5 (275.5–745.3)	621.0 (491.5–845.5) *p* = 0.1823	-	269.0 (244.5–394.0)	530.0 (432.3–667.8) *p* = 0.0062	1.245 (0.975–1.589) *p* = 0.080
***Immune cell-related blood indices***						
Neutrophil count (10^9^/L)	8.07 (4.20–14.63)	15.84 (12.68–19.92) *p* = 0.0101	1.364 (1.017–1.829) *p* = 0.038	7.07 (5.96–7.91)	13.78 (11.00–17.67) *p* = 0.0016	1.003 (0.523–1.925) *p* = 0.992
NLR	12.84 (4.02–24.96)	28.44 (16.24–55.54) *p* = 0.0279	1.106 (0.987–1.239) *p* = 0.083	7.97 (4.79–11.78)	20.55 (11.84–42.40) *p* = 0.0295	1.242 (0.937–1.646) *p* = 0.132
IL-6 (pg/mL)	7.60 (4.35–21.15)	46.90 (10.90–87.35) *p* = 0.0500	1.054 (0.883–1.257) *p* = 0.561	3.40 (2.40–15.10)	74.40 (10.90–182.00) *p* = 0.0667	-

Inflammatory-immune biomarkers showing statistically significant differences between survivors and non-survivors (by sex) were evaluated using univariate logistic regression, with COVID-19 mortality as the dependent variable. Data are reported as odds ratios (OR) with 95% confidence intervals (CI). The OR scales are defined as follows: CRP per 10 mg/L increase; LDH per 10 U/L increase; IL-6 per 10 pg/mL increase; D-dimer per 0.1 mg/L increase; neutrophil count per 10^9^/L increase; and NLR per 1-unit increase. *p* ≤ 0.05 indicates statistical significance. Abbreviations: IQR, interquartile range; CRP, C-reactive protein; LDH, lactate dehydrogenase; NLR, neutrophil-to-lymphocyte ratio; IL, interleukin.

**Table 7:** Receiver operating characteristic (ROC) analysis of CRP and neutrophil count for predicting death in severe-to-critical male COVID-19 patients on day 7 after admission.

	Males			
**On day 7** **after admission**	AUC (95% CI)	Sensitivity (%)	Specificity (%)	Cut-off
***Acute-phase*** ***protiens***				
CRP (mg/L)	0.856 (0.670–1) *p* = 0.009	77.8	90	39.25
***Immune cell-related*** ***blood indices***				
Neutrophil count (10^9^/L)	0.844 (0.668–1) *p* = 0.011	100	60	11.03

Data are presented as area under the curve (AUC) and 95% confidence interval (CI). *p* ≤ 0.05 was considered statistically significant. Cut-off value were determined as described in the Materials and Methods section. Abbreviation: CRP, C-reactive protein.

## Data Availability

The original contributions presented in this study are included in the article/Supplementary Material. Further inquiries can be directed to the corresponding author.

## Supplementary Materials

The following supporting information can be downloaded at: https://www.mdpi.com/article/10.3390/medsci14020282/s1, Table S1. Inflammatory and immune-cell based blood indices in healthy males and females; Figure S1. Acute-phase proteins in males and females with different COVID-19 severity at admission and on day 7 after admission; Table S2. Receiver operating characteristic (ROC) analyses of independent predictors combined in multivariable models predicting COVID-19 severity by sex at admission; Table S3. Receiver operating characteristic (ROC) analyses of independent predictors combine in multivariable model predicting COVID-19 severity in males on day 7 after admission; Figure S2. Immune cell-related indices in males and females with different COVID-19 severity at admission and on day 7 after admission; Table S4. Prevalence of IL-6, IL-10, and IL-17 by COVID-19 severity, sex, and admission time; Table S5. Receiver operating characteristic (ROC) analyses of independent predictors combined in multivariable model predicting COVID-19 severity in males at admission; Figure S3. Acute-phase proteins in severe-to-critical males and female COVID-19 subjects with different survival outcome at admission and on day 7 after admission; Figure S4. Immune cell-related indices in severe-to-critical males and female COVID-19 subjects with different survival outcome at admission and on day 7 after admission; Table S6. Prevalence of IL-6, IL-10, and IL-17 in severally-to-critically ill COVID-19 by survival outcome, sex, and admission time.
