# Supplementary material for: Sex-Specific Signatures of Circulating Protein and Cellular Host Responses Predicting COVID-19 Severity
Source: Med Sci (Basel). 2026 May 31;14(2):282. doi: 10.3390/medsci14020282 (PMC13302944; doi:10.3390/medsci14020282)
Supplement: Supplementary file 1 [file medsci-14-00282-s001.zip › Figure S1.pdf]

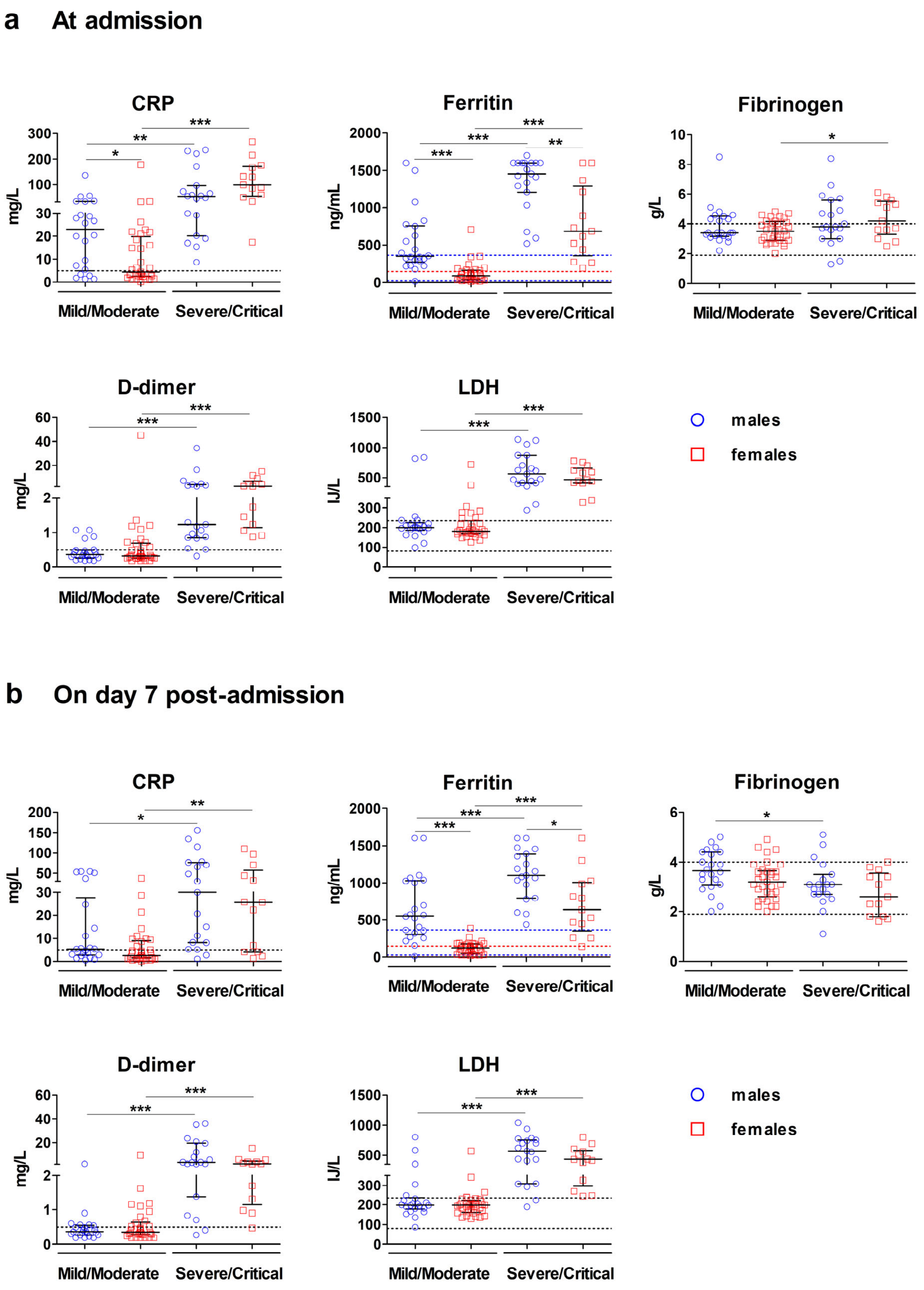


**Figure S1. Acute-phase proteins in males and females with different COVID-19 severity at admission and on day 7 post-admission.** Scatter dot plots display blood levels of CRP, ferritin, fibrinogen, D-dimer, and LDH in male and female COVID-19 patients with mild-to-moderate and severe-to-critical disease (a) at admission and (b) on day 7 post-admission. Dashed lines on plots denote reference ranges for specific variables. Data are presented as median and interquartile range. $p \leq 0.05$ indicates statistical significance. $^{*}$ $p \leq 0.05$; $^{**}$ $p \leq 0.01$; $^{***}$ $p \leq 0.001$. Abbreviations: CRP, C-reactive protein; LDH, lactate dehydrogenase.
