# Supplementary material for: Sex-Specific Signatures of Circulating Protein and Cellular Host Responses Predicting COVID-19 Severity
Source: Med Sci (Basel). 2026 May 31;14(2):282. doi: 10.3390/medsci14020282 (PMC13302944; doi:10.3390/medsci14020282)
Supplement: Supplementary file 1 [file medsci-14-00282-s001.zip › Figure S2.pdf]

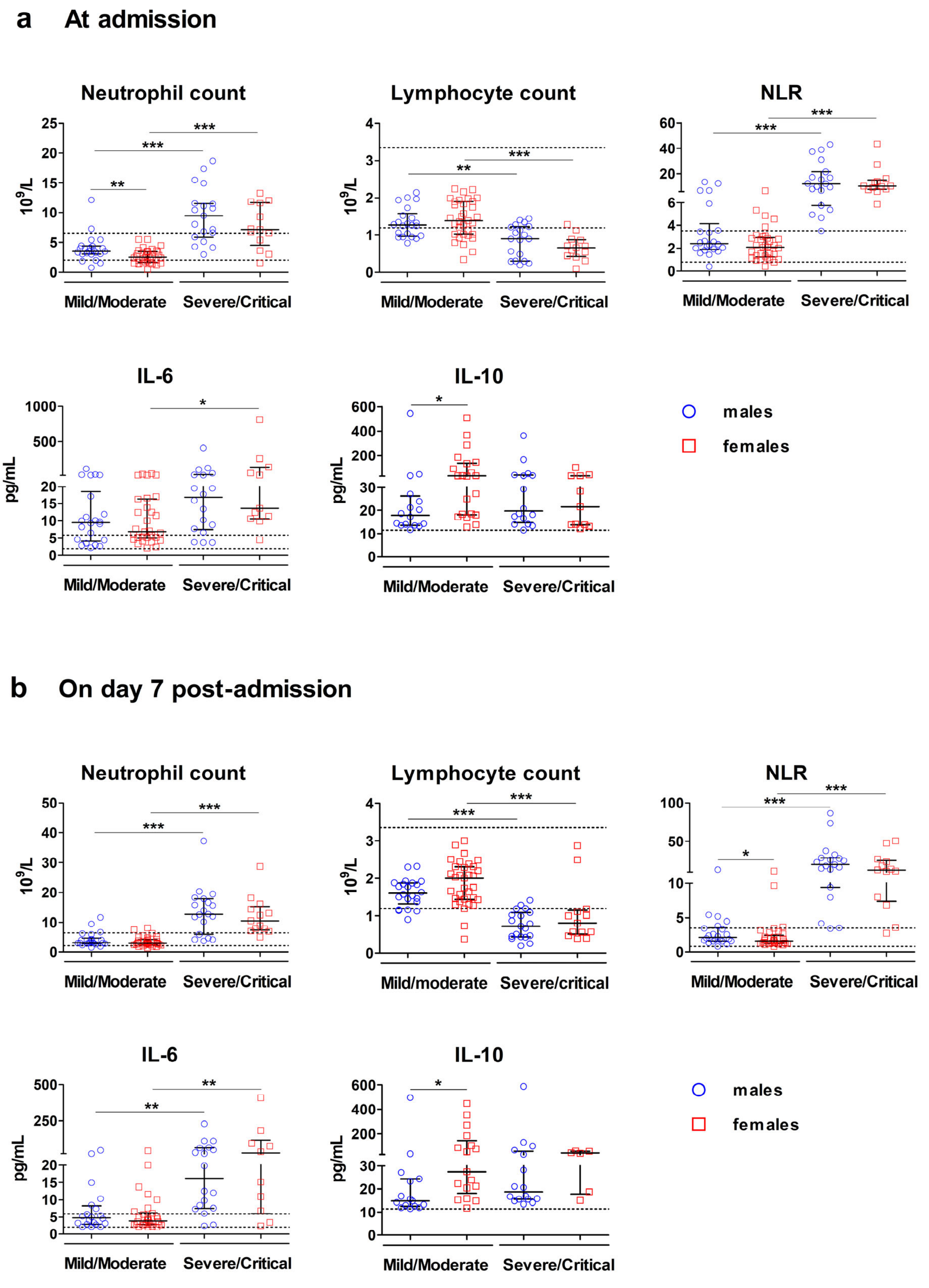

Figure S2. Immune cell-related indices in males and females with different COVID-19 severity at admission and on day 7 post-admission. Scatter dot plots display neutrophil and lymphocyte counts, NLR, IL-6, and IL-10 blood levels in male and female COVID-19 patients with mild-to-moderate and severe-to-critical disease (a) at admission and (b) on day 7 post-admission. Dashed lines on plots denote reference ranges for specific variables. Data are presented as median and interquartile range. $p \leq 0.05$ indicates statistical significance. * $p \leq 0.05$; ** $p \leq 0.01$; *** $p \leq 0.001$. Abbreviations: NLR, neutrophil to lymphocyte ratio; IL, interleukin.
