# Supplementary material for: Sex-Specific Signatures of Circulating Protein and Cellular Host Responses Predicting COVID-19 Severity
Source: Med Sci (Basel). 2026 May 31;14(2):282. doi: 10.3390/medsci14020282 (PMC13302944; doi:10.3390/medsci14020282)
Supplement: Supplementary file 1 [file medsci-14-00282-s001.zip › Figure S3.pdf]

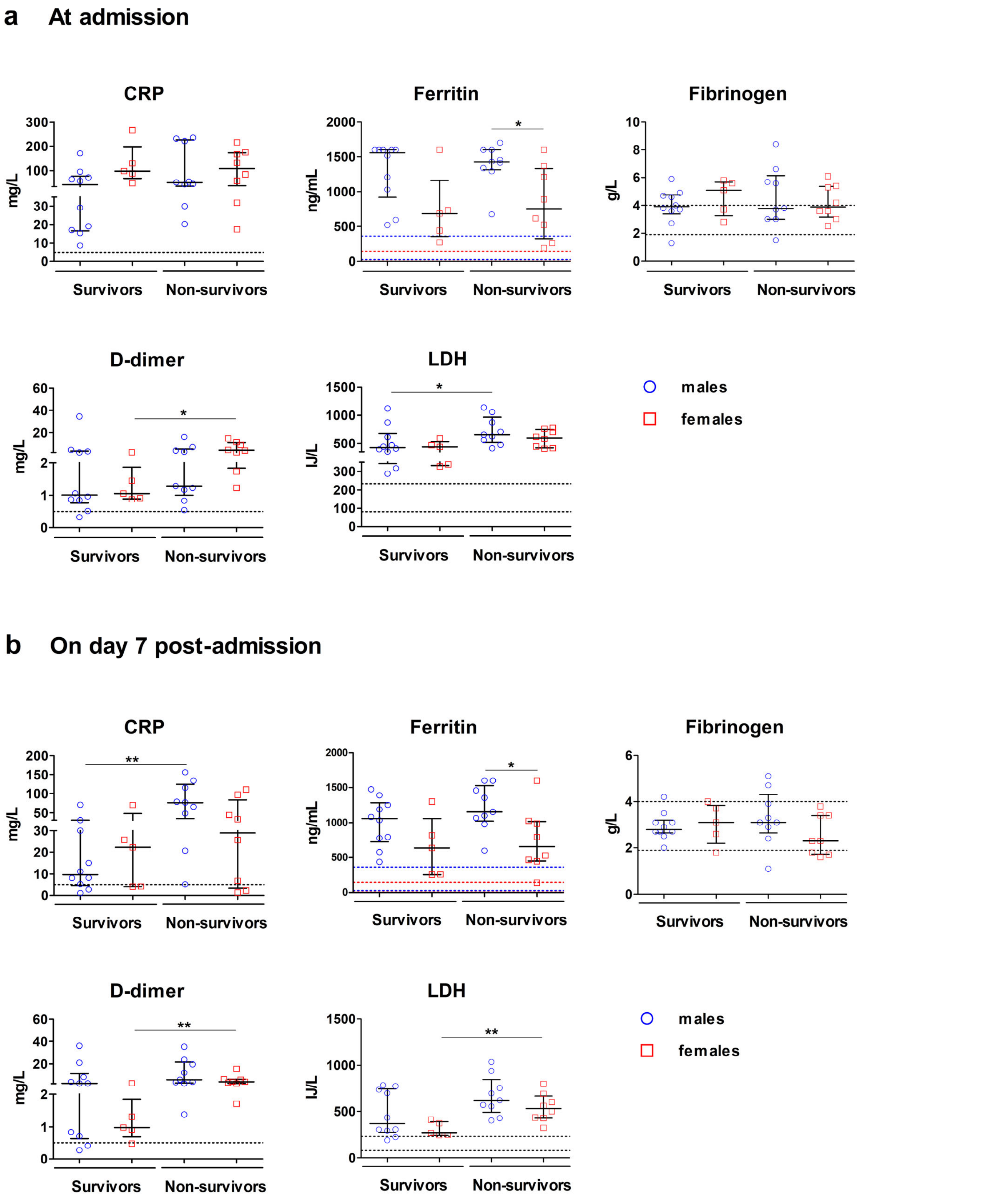

Figure S3. Acute-phase proteins in severe-to-critical males and female COVID-19 subjects with different survival outcome at admission and on day 7 post-admission. Scatter dot plots display blood levels of CRP, ferritin, fibrinogen, D-dimer, and LDH in severe-to-critical male and female COVID-19 patients stratified by survival outcome (a) at admission and (b) on day 7 post-admission. Dashed lines on plots denote reference ranges for specific variables. Data are presented as median and interquartile range. $p \leq 0.05$ indicates statistical significance. * $p \leq 0.05$; ** $p \leq 0.01$; *** $p \leq 0.001$. Abbreviations: CRP, C-reactive protein; LDH, lactate dehydrogenase
