# Supplementary material for: Sex-Specific Signatures of Circulating Protein and Cellular Host Responses Predicting COVID-19 Severity
Source: Med Sci (Basel). 2026 May 31;14(2):282. doi: 10.3390/medsci14020282 (PMC13302944; doi:10.3390/medsci14020282)
Supplement: Supplementary file 1 [file medsci-14-00282-s001.zip › Table S1.pdf]

Table S1. Inflammatory and immune-cell based blood indices in healthy males and females.

**Parameter**	**Healthy controls (n = 10)**		**Laboratory Reference Ranges in Healthy Adults KBC "Dr Dragiša Mišović - Dedinje"**
	Males (n = 5)	***Females (n = 5)***	
CRP	0.40 (0.40 - 2.65)	1.20 (0.85 - 1.80)	< 5.0 mg/L
Ferritin	75.24 ± 21.45	37.00 ± 25.21<br>p = 0.032	Males: 28 - 365 ng/mL<br>Females: 5 - 148 ng/mL
Fibrinogen	3.00 ± 0.70	3.22 ± 0.37	1.9 - 4.0 g/L
D-dimer	0.280 (0.250 - 0.525)	0.300 (0.270 - 0.395)	0 - 0.50 mg/L
LDH	170.40 ± 29.64	159.40 ± 13.16	81 - 234 IJ/L
Neutrophil counts	3.696 (3.221 - 4.418)	3.753 (2.543 - 4.999)	2.06 - 6.49 10^9/L
Lymphocyte counts	1.97 ± 0.44	2.40 ± 1.01	1.19 - 3.35 10^9/L
NLR	1.580 (1.371 - 2.264)	1.783 (1.385 - 2.375)	0.78 - 3.53
IL-6	2.56 ± 0.50	2.80 ± 0.80	2 - 5.9 pg/mL

Data are presented as mean ± standard deviation or as median (interquartile range). $p \leq 0.05$ was considered statistically significant. Notably, the differences between sexes were not statistically significant, except for ferritin. Abbreviations: CRP, C-reactive protein; LDH, lactate dehydrogenase; NLR, neutrophil-to-lymphocyte ratio; IL, interleukin.
