# Supplementary material for: Sex-Specific Signatures of Circulating Protein and Cellular Host Responses Predicting COVID-19 Severity
Source: Med Sci (Basel). 2026 May 31;14(2):282. doi: 10.3390/medsci14020282 (PMC13302944; doi:10.3390/medsci14020282)
Supplement: Supplementary file 1 [file medsci-14-00282-s001.zip › Table S2.pdf]

Table S2. Receiver operating characteristic (ROC) analyses of independent predictors combined in multivariable models predicting COVID-19 severity by sex at admission.

Acute-phase proteins	At admission			
	AUC (95% CI)	Sensitivity (%)	Specificity (%)	Cut-off
Male sub-cohorts				
Ferritin supra cut-off levels (≥929 ng/mL):				
D-dimer (mg/L)	0.958 (0.862-1) p = 0.014	87.5	100	0.84
D-dimer supra cut-off levels (≥0.505 mg/L):				
Ferritin (ng/mL)	0.819 (0.597-1) p = 0.050	88.9	75	903.5
Female sub-cohorts				
Ferritin supra cut-off levels (≥191 ng/mL):				
D-dimer (mg/L)	0.892 (0.734-1) p = 0.012	69.2	100	1.40
D-dimer supra cut-off levels (≥0.84 mg/L):				
Ferritin (ng/mL)	0.908 (0.764-1) p = 0.009	76.9	100	395.5

Sequential ROC analyses of the predictive capacity of ferritin and D-dimer in models combining these two inflammatory blood indices were performed in male and female sub-cohorts with supra cut-off levels of D-dimer and ferritin, respectively. Ferritin and D-dimer cut-off values for male and female COVID-19 subjects are displayed in Table 3. Data are presented as area under the curve (AUC) and 95% confidence interval (CI). $p \leq 0.05$ was considered statistically significant. The cut-off values were determined as described in the Materials and Methods section.
