# Supplementary material for: Sex-Specific Signatures of Circulating Protein and Cellular Host Responses Predicting COVID-19 Severity
Source: Med Sci (Basel). 2026 May 31;14(2):282. doi: 10.3390/medsci14020282 (PMC13302944; doi:10.3390/medsci14020282)
Supplement: Supplementary file 1 [file medsci-14-00282-s001.zip › Table S3.pdf]

Table S3. Receiver operating characteristic (ROC) analyses of independent predictors combine in multivariable model predicting COVID-19 severity in males on day 7 post-admission.

Acute-phase proteins	On day 7 post-admission AUC (95% CI)	Sensitivity (%)	Specificity (%)	Cut-off
Male sub-cohorts				
D-dimer supra cut-off levels (≥ 0.65 mg/L):				
Ferritin (ng/mL)	0.735 (0.505-0.966) $p = 0.288$	64.7	100	1017
Fibrinogen (g/L)	0.838 (0.660-1) $p = 0.127$	76.5	100	3.7
Ferritin supra cut-off levels (≥ 575 ng/mL):				
D-dimer (mg/L)	0.873 (0.736-1) $p = 0.002$	83.3	88.9	1.14
Fibrinogen (g/L)	0.920 (0.815-1) $p < 0.001$	66.7	100	3.2
Fibrinogen sub cut-off levels (≤ 3.2 g/L):				
D-dimer (mg/L)	0.974 (0.912-1) $p = 0.001$	92.3	100	0.375
Ferritin (ng/mL)	0.987 (0.947-1) $p = 0.001$	92.3	100	571

Sequential ROC analyses of the predictive capacity of ferritin and fibrinogen, D-dimer and fibrinogen, and D-dimer and ferritin were performed in male sub-cohorts with supra optimal cut-off blood levels of D-dimer, and ferritin and sub optimal cut-off levels of fibrinogen, respectively. D-dimer and ferritin cut-off values for male COVID-19 subjects are displayed in Table 3. Data are presented as area under the curve (AUC) and 95% confidence interval (CI). $p \leq 0.05$ was considered statistically significant. The cut-off values were determined as described in the Materials and Methods section.
