# Supplementary material for: Sex-Specific Signatures of Circulating Protein and Cellular Host Responses Predicting COVID-19 Severity
Source: Med Sci (Basel). 2026 May 31;14(2):282. doi: 10.3390/medsci14020282 (PMC13302944; doi:10.3390/medsci14020282)
Supplement: Supplementary file 1 [file medsci-14-00282-s001.zip › Table S4.pdf]

Table S4. Prevalence of IL-6, IL-10, and IL-17 by COVID-19 severity, sex and admission time.

**At admission**	**n**	**IL-6 ≥ 2 pg/mL n (%)**	**IL-10 ≥ 11.5 pg/mL n (%)**	**IL-17 ≥ 8.05 pg/mL n (%)**
Males	41	40 (97.6%)	33 (80.5%)	13 (31.7%)
Mild/Moderate	22	22 (100%)	17 (77.3%)	6 (27.3%)
Severe/Critical	19	18 (94.7%)	16 (84.2%)	7 (36.8%)
Females	46	41 (89.1%)	32 (69.6%)	17 (37.0%)
Mild/Moderate	33	30 (90.9%)	21 (63.6%)	13 (39.4%)
Severe/Critical	13	11 (84.6%)	11 (84.6%)	4 (30.8%)
**On day 7 post-admission**				
Males	41	38 (92.7%)	31 (75.6%)	NA
Mild/Moderate	22	20 (90.9%)	15 (68.2%)	NA
Severe/Critical	19	18 (94.7%)	16 (84.2%)	NA
Females	46	40 (86.9%)	23 (50.0%)	NA
Mild/Moderate	33	30 (90.9%)	17 (51.5%)	NA
Severe/Critical	13	10 (76.9%)	6 (46.2%)	NA

Data are presented as integers and percentages. IL, interleukin; NA, not applicable.
