# Supplementary material for: Sex-Specific Signatures of Circulating Protein and Cellular Host Responses Predicting COVID-19 Severity
Source: Med Sci (Basel). 2026 May 31;14(2):282. doi: 10.3390/medsci14020282 (PMC13302944; doi:10.3390/medsci14020282)
Supplement: Supplementary file 1 [file medsci-14-00282-s001.zip › Table S5.pdf]

Table S5. Receiver operating characteristic (ROC) analyses of independent predictors combined in multivariable model predicting COVID-19 severity in males at admission.

**Immune cell-based blood indices**	**At admission** AUC (95% CI)	Sensitivity (%)	Specificity (%)	Cut-off
Male sub-cohorts				
Lymphocytes sub cut-off levels (≤ 0.9 10^9/L):				
Neutrophil count (10^9/L)	0.850 (0.579-1) p = 0.133	70	100	5.65
Neutrophils supra cut-off levels (≥ 5.67 10^9/L):				
Lymphocyte count (10^9/L)	0.533 (0.274-0.793) p = 0.881	46.7	100	0.93

Sequential ROC analyses of the predictive capacity of neutrophil and lymphocyte count in model combining these two immune cell-based blood indices were performed in male sub-cohorts with sub and supra optimal cut-off levels of lymphocytes and neutrophils, respectively. Neutrophil and lymphocyte cut-off values for male COVID-19 subjects are displayed in Table 5. Data are presented as area under the curve (AUC) and 95% confidence interval (CI). $p \leq 0.05$ was considered statistically significant. The cut-off values were determined as described in the Materials and Methods section.
