# Supplementary material for: Sex-Specific Signatures of Circulating Protein and Cellular Host Responses Predicting COVID-19 Severity
Source: Med Sci (Basel). 2026 May 31;14(2):282. doi: 10.3390/medsci14020282 (PMC13302944; doi:10.3390/medsci14020282)
Supplement: Supplementary file 1 [file medsci-14-00282-s001.zip › Table S6.pdf]

Table S6. Prevalence of IL-6, IL-10, and IL-17 in severally-to-critically ill COVID-19 by survival outcome, sex, and admission time.

**At admission**	**n**	**IL-6 ≥ 2 pg/mL n (%)**	**IL-10 ≥ 11.5 pg/mL n (%)**
Males	19	18 (94.7%)	16 (84.2%)
Survivors	10	9 (90.0%)	8 (80.0%)
Non-survivors	9	9 (100%)	8 (88.9%)
Females	13	11 (84.6%)	11 (84.6%)
Survivors	5	3 (60.0%)	4 (80.0%)
Non-survivors	8	8 (72.7%)	7 (87.5%)
**On day 7 post-admission**			
Males	19	18 (94.7%)	16 (84.2%)
Survivors	10	9 (90.0%)	8 (80.0%)
Non-survivors	9	9 (100%)	8 (88.9%)
Females	13	10 (76.9%)	6 (46.2%)
Survivors	5	3 (60.0%)	0
Non-survivors	8	7 (87.5%)	6 (75.0%)

Data are presented as integers and percentages. IL, interleukin.
